# Stapled BIG3 helical peptide ERAP potentiates anti-tumour activity for breast cancer therapeutics

**DOI:** 10.1038/s41598-017-01951-6

**Published:** 2017-05-12

**Authors:** Tetsuro Yoshimaru, Keisuke Aihara, Masato Komatsu, Yosuke Matsushita, Yasumasa Okazaki, Shinya Toyokuni, Junko Honda, Mitsunori Sasa, Yasuo Miyoshi, Akira Otaka, Toyomasa Katagiri

**Affiliations:** 1 Division of Genome Medicine, Institute for Genome Research, Tokushima University, Tokushima, 770-8503 Japan; 2 Institute of Health Biosciences and Graduate School of Pharmaceutical Sciences, Tokushima University, Tokushima, 770-8505 Japan; 3 Department of Pathology and Biological Responses, Nagoya University Graduate School of Medicine, Aichi, 466-8550 Japan; 4 Department of Surgery, National Hospital Organization Higashitokushima Medical Center, Tokushima, 779-0193 Japan; 5 Department of Surgery, Tokushima Breast Care Clinic, Tokushima, 770-0052 Japan; 6 Department of Surgery, Division of Breast and Endocrine Surgery, Hyogo College of Medicine, Hyogo, 663-8501 Japan

## Abstract

Estradiol (E2) and the oestrogen receptor-alpha (ERα) signalling pathway play pivotal roles in the proliferative activity of breast cancer cells. Recent findings show that the brefeldin A-inhibited guanine nucleotide-exchange protein 3-prohibitin 2 (BIG3-PHB2) complex plays a crucial role in E2/ERα signalling modulation in breast cancer cells. Moreover, specific inhibition of the BIG3-PHB2 interaction using the ERα activity-regulator synthetic peptide (ERAP: 165–177 amino acids), derived from α-helical BIG3 sequence, resulted in a significant anti-tumour effect. However, the duration of this effect was very short for viable clinical application. We developed the chemically modified ERAP using stapling methods (stapledERAP) to improve the duration of its antitumour effects. The stapledERAP specifically inhibited the BIG3-PHB2 interaction and exhibited long-lasting suppressive activity. Its intracellular localization without the membrane-permeable polyarginine sequence was possible via the formation of a stable α-helix structure by stapling. Tumour bearing-mice treated daily or weekly with stapledERAP effectively prevented the BIG3-PHB2 interaction, leading to complete regression of E2-dependent tumours *in vivo*. Most importantly, combination of stapledERAP with tamoxifen, fulvestrant, and everolimus caused synergistic inhibitory effects on growth of breast cancer cells. Our findings suggested that the stapled ERAP may be a promising anti-tumour drug to suppress luminal-type breast cancer growth.

## Introduction

Breast cancer is the most common cancer among women, and its incidence is rising worldwide. Approximately 70% of breast cancer cells express oestrogen receptor-alpha (ERα) and depend on estradiol (E2) for growth and survival. Highly effective therapeutics are currently being used to block the E2/ERα signalling pathway, such as selective ERα modulators (e.g. tamoxifen and raloxifene), ERα down-regulators (e.g. fulvestrant), and the aromatase inhibitor (AI)^1–3^. However, their effectiveness is limited because of the high rate of intrinsic and acquired endocrine resistance^4–7^. The precise molecular mechanism(s) governing resistance in luminal-type breast cancer is an active area of research. Identifying the factors and pathways responsible for resistance and developing novel therapies to treat breast cancer are crucial. BIG3 (brefeldin A-inhibited guanine nucleotide-exchange protein 3) is exclusively overexpressed in the majority of breast cancers^8, 9^, and it interacts with the tumour suppressor PHB2 (prohibitin 2) in the cytoplasm, thereby inhibiting E2-dependent translocation to the nucleus and plasma membrane. This interaction results in the constitutive activation of the E2/ERα signalling pathway in breast cancer cells^9^.

We previously developed a dominant-negative peptide, ER α activity-regulator synthetic peptide (ERAP), which specifically disrupts the BIG3-PHB2 interaction and inhibits multiple ERα-signalling pathways driving breast cancer cell growth by reactivating tumour-suppressive activity of PHB2^10^. Cell-based assays and mouse xenograft studies showed that ERAP completely suppressed E2-dependent breast cancer cell growth *in vitro* and *in vivo*, respectively^10^. However, its inhibitory effect was only maintained for 24 h, most likely due to its high susceptibility to proteolytic degradation. Hence, we aimed to improve the proteolytic stability of ERAP to better maintain its inhibitory activity through a hydrocarbon-stapling strategy. Recent cancer therapy studies have shown that stapled peptides can modulate intracellular and extracellular protein–protein interactions^11^.

In the present study, stapled ERAP (stERAP), possessing stabilized α-helices, sustained inhibition of the BIG3-PHB2 interaction, thereby suppressing constitutive ERα activation in breast cancer cells. In addition, stERAP caused complete regression of E2-dependent tumour formation without any detectable toxicity in murine xenografts. These findings suggest that hydrocarbon stapling of ERAP may provide an effective therapeutic strategy to modulate the BIG3-PHB2 interaction in E2-dependent breast cancer cells and may be a viable means to overcome endocrine resistance.

## Results

### Screening stapled α-helical ERAP

**Figure 1:**
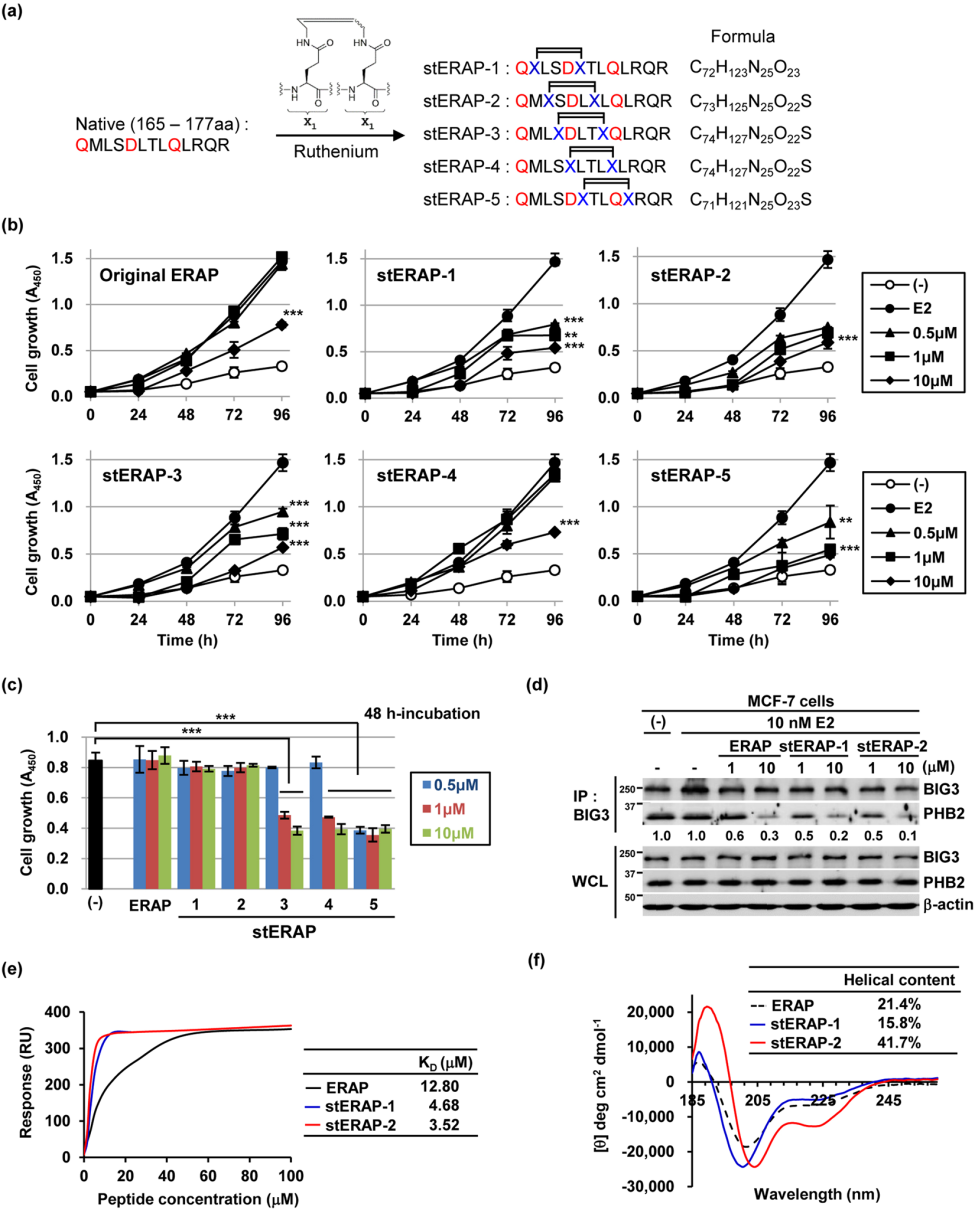
Stapled ERAP has sustained stability and highly sensitive capacity to inhibit the BIG3-PHB2 interaction. (**a**) Primary structures for ERAP and its stapled analogs. (**b**,**c**) An MTT assay evaluating the inhibitory effects of stapled ERAPs on the growth of 10 nM E2-dependent MCF-7 cells (**b**) and of mammary epithelial MCF-10A cells (**c**). Cells were given a single treatment at 0 h. These data represent the mean ± s.d. of three independent experiments (**P < 0.01 and ***P < 0.001 via two-sided Student’s *t*-tests). (**d**) The inhibitory effects of stapled ERAP treatment on BIG3-PHB2 interactions in MCF-7 cells. ERAP was used as a positive control for the inhibition of the BIG3-PHB2 interaction. (**e**) *In vitro* direct interaction of stapled ERAPs (stERAP-1 and -2) and recombinant PHB2 discerned by surface plasmon resonance systems. (**f**) CD spectra and α-helical content of ERAP and stERAP analogs (stERAP-1 and -2) in 10 mM sodium phosphate buffer (pH 7.0).

We aimed to chemically modify ERAP (amino acids 165–177) on the PHB2 binding α-helices of BIG3 using hydrocarbon stapling. In particular, we employed olefin metathesis to enhance ERAP’s stability regarding its inhibition of the BIG3-PHB2 interaction. We synthesized a series of stapled α-helices of ERAP bearing the olefin scaffold (stERAP-1 to -5) (Fig. 1a). Notably, 165Q, 169D, and 173Q amino acids within the ERAP sequence are on the α-helical surface and are critical for its interaction with PHB2 (ref. 10). Therefore, except in stERAP-4, these critical residues were maintained, and amino acid (X) for stapling was incorporated in an *i* to *i* + 4 relationship to display the 165Q, 169D, and 173Q residues via α-helix formation. The resulting stERAPs were then screened using a cell-based assay. Treatment of stERAP-1, -2, -3, and -5 significantly reduced E2-dependent cell growth in a dose-dependent manner (IC_50_ = 0.89 μM, 1.02 μM, 0.81 μM, and 0.68 μM, respectively) at 96 h after stERAP and E2 treatments, but unstapled original ERAP did not reduce E2-dependent cell growth until after 96 h (IC_50_ = 7.97 μM; Fig. 1b and Supplementary Fig. S1a). stERAP-4, which lacked the 169D and 173Q residues, possessed only one of the critical residues and showed no dose-dependent inhibition of E2-dependent cell growth at 96 h (IC_50_ = 7.89 μM; Fig. 1b and Supplementary Fig. S1a). Notably, stERAP-1 and -2, but not -3, -4, and -5, had no significant effect on the growth of normal mammary epithelial MCF-10A cells, which do not express ERα and BIG3 (Fig. 1c and Supplementary Fig. S1a,b). This suggested that stERAP-1 and -2 might specifically inhibit the growth of ERα and BIG3-positive breast cancer cells. To clarify the off-target effects of stERAP-5 on the growth of normal cells, we performed a DNA microarray of MCF-10A cells 24 and 48 h after treatment of stERAP-2 and -5, respectively. Analyses of gene expression profiles identified 93 and 191 transcripts that were up- or down-regulated, respectively, with >100-fold in cells of 48 h post treatment with stERAP-5 compared with cells treated with stERAP-2 (Supplementary Fig. S1c and SupplementaryTable S1). However, only four genes were differentially expressed between stERAP-2- and -5-treated cells at 24 h post treatment (Supplementary Fig. S1c and SupplementaryTable S2). Gene annotation enrichment analysis of 284 genes using the DAVID (The Database for Annotation, Visualization and Integrated Discovery) algorithm and GeneMANIA software revealed a prominent subset of extracellular matrix-associated genes (Supplementary Fig. S1d), suggesting that stERAP-5 has potential off-target effects on extracellular matrix-related pathways that affect normal epithelial cell growth. Accordingly, we focused on stERAP-1 and -2 for further analyses.

We next investigated whether stERAP-1 and -2 inhibited the BIG3-PHB2 interaction by co-immunoprecipitation experiments with an anti-BIG3 antibody. The results showed that these stERAPs dose-dependently inhibited the endogenous BIG3-PHB2 interaction in MCF-7 cells similar to that of unstapled original ERAP (Fig. 1d). Subsequently, we examined the affinity of these stERAPs to His-tagged recombinant PHB2 (His-PHB2) by surface plasmon resonance interaction analysis. stERAP-1 and -2 showed an approximately three-fold enhancement in binding affinity (4.68 and 3.52 μM, respectively) compared with that of the unstapled, original ERAP (12.80 μM; Fig. 1e). We performed CD spectroscopy to investigate the conformational properties of stERAP-1 and -2 and found that stERAP-2 had a higher α-helical content (41.7%) than that of the unstapled original ERAP and stERAP-1 (21.4% and 15.8%, respectively), indicating the enhanced stabilization of the α-helical structure in stERAP-2 (Fig. 1f). We confirmed that stERAP-2 significantly suppressed the E2-induced expression of the ERα-target genes *TFF1* and *CCND1* for 96 h treatment (Supplementary Fig. S1e). Taken together, these findings suggested that the high α-helical content of stERAP-2 enhanced proteolytic stability, which may be strongly correlated with sustained suppression of breast cancer cell growth.

### *In vitro* anti-proliferative activity of stERAP without olefin linkage on E2-dependent breast cancer cells

**Figure 2:**
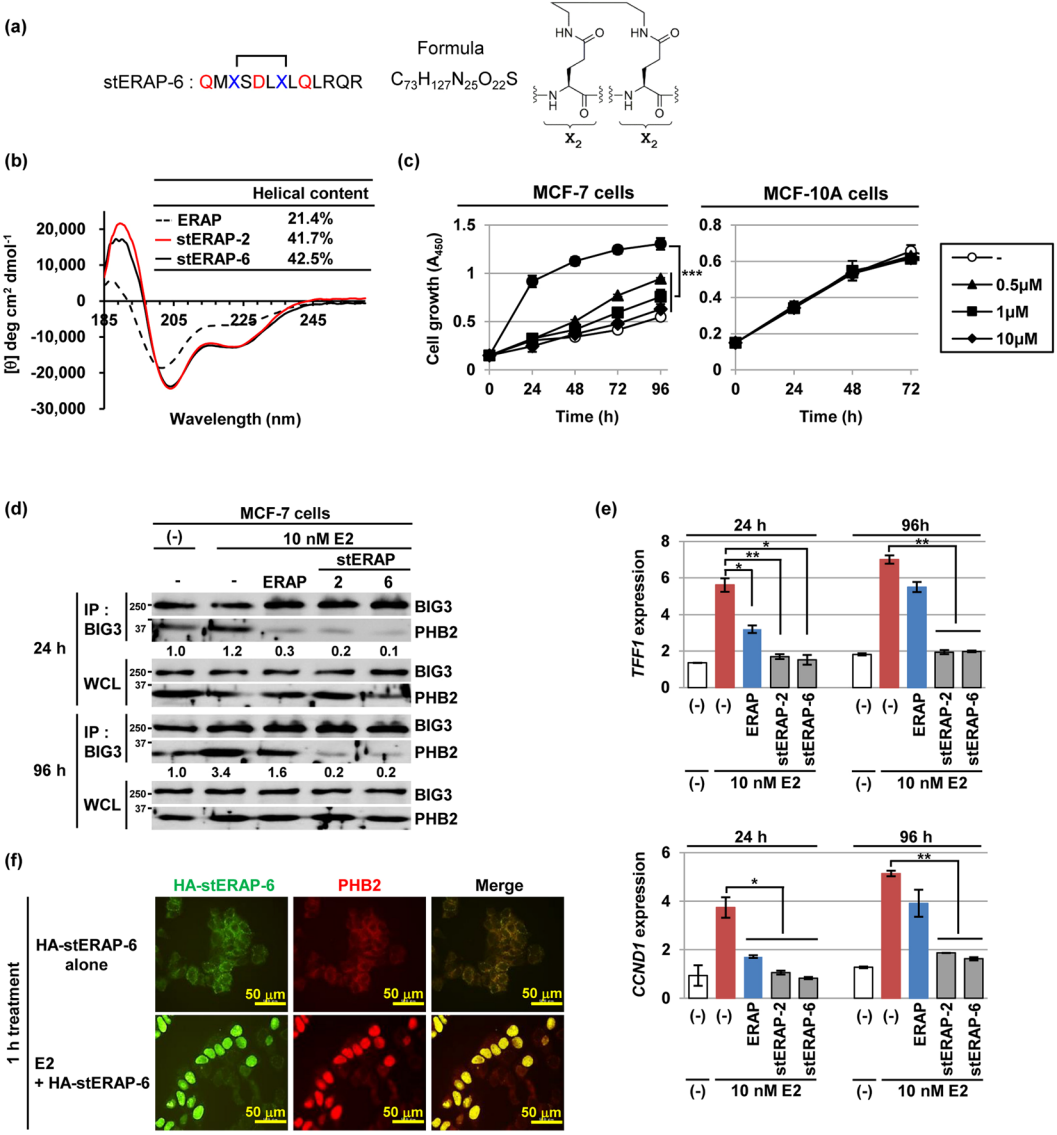
Stapled ERAP without olefin metathesis suppresses the E2-dependent responsiveness with long-term stability. (**a**) Primary structures for stapled ERAP (stERAP-6) without olefin. (**b**) CD spectra and α-helical content of stERAP-6. (**c**) An MTT assay evaluating the duration of the inhibitory effects of stERAP-6 on the growth of 10 nM E2-dependent MCF-7 cells (left) and of mammary epithelial MCF-10A cells (right). Cells were given a single treatment at 0 h. These data represent the mean ± s.d. of three independent experiments. (**d**) The inhibitory effects of stERAP-6 on BIG3-PHB2 interactions in MCF-7 cells. (**e**) The duration of the inhibitory effects of stERAP-6 on ERα-target genes expression in MCF-7 cells for the times indicated. The results were expressed as the fold increase over untreated cells at 24 h (set at 1.0). The data represent the mean ± s.e.m. of three independent experiments. (**f**) Representative immunofluorescence images of the subcellular localization of HA-tagged stERAP-6 (HA-stERAP-6; green) and PHB2 (red) in the presence of E2. *P < 0.05, **P < 0.01, and ***P < 0.001 via two-sided Student’s *t*-tests.

The ruthenium-catalyzed olefin metathesis employed for the preparation of stERAP-2 has gained in popularity with regard to hydrocarbon stapling; however, the use of the ruthenium catalyst increases the cost of peptide preparation and may complicate the removal of the metal catalyst. Because the saturated hydrocarbon-stapled peptide that is obtained by reduction of the olefin unit of stERAP-2, exhibits suppressive activity comparable to that of stERAP-2, stapling using not only olefin but also saturated hydrocarbon structures seemed to be attributable to sustained inhibitory activity. This hypothesis prompted us to use a stapling protocol alternative to olefin metathesis, particularly an intramolecular amidation reaction between glutamic acid derivatives (Fig. 2a). The alternative stapled ERAP (stERAP-6) prepared by the amidation protocol maintained 42.5% helicity, which was similar to that of stERAP-2 (41.7%; Fig. 2b), and resulted in prolonged inhibition of E2-dependent proliferation of MCF-7 cells for 96 h (Fig. 2c). This was not observed for MCF-10A cells (Fig. 2c). Co-immunoprecipitation with BIG3 antibody and qRT-PCR analyses indicated that treatment with stERAP-6, but not with unstapled original ERAP, led to effective inhibition of the endogenous BIG3-PHB2 formation and downregulation of ERα-target genes *TFF1* and *CCND1* until 96 h (Fig. 2d,e), suggesting the possibility that this prolonged anti-proliferative effect is due to the high PHB2-binding ability of stERAPs compared with unstapled original ERAP (Fig. 1e). We next investigated the subcellular distribution of HA-tagged stERAP-6 (HA-stERAP-6). As expected, in the presence of E2, HA-stERAP-6 was nuclear-translocated with endogenous PHB2 even after 1 h (Fig. 2f). Consequently, HA-stERAP-6 significantly suppressed E2-dependent MCF-7 cell growth (Supplementary Fig. S2a,b). These results suggest that stERAP-6 possessed cell-membrane permeability and growth-suppressive activity in MCF-7 cells, although this stapled peptide lacked cell-permeable polyarginine residues.

### *In vivo* efficacy of stERAP-6

**Figure 3:**
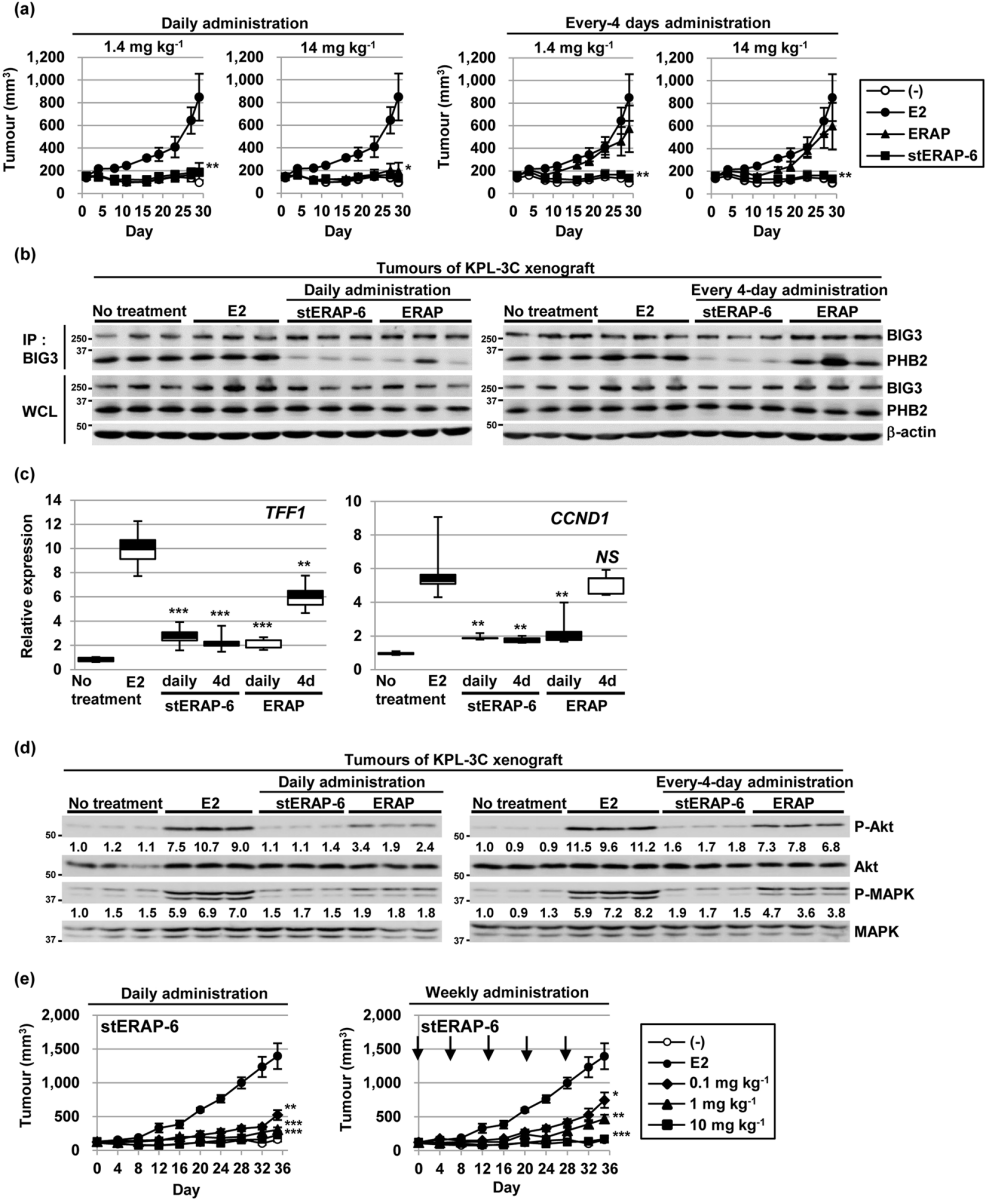
StERAP-6 has *in vivo* anti-tumour efficacy in xenograft models of human ERα-positive breast cancer. (**a**) Tumour growth assessed after daily (left) or every 4 days (right) intraperitoneal injection of 1.4 and 14 mg kg^−1^ stERAP-6 or unstapled original ERAP (ERAP) in KPL-3C xenograft mice. The tumour sizes represent the mean ± s.e.m. of each group (n = 5). (**b**) Immunoblot analysis of the binding inhibition of the BIG3-PHB2 interaction in tumours treated intraperitoneally with daily (left) or every 4 days (right) of stERAP-6 or ERAP. (**c**) The inhibitory effects of stERAP-6 or ERAP on ERα-target genes expression in tumours treated for 28 days. The results were expressed as the fold increase over untreated tumours (set at 1.0). *NS:* no significance. The data represent the mean ± s.e.m. of five independent tumours. (**d**) Immunoblot analysis of the phosphorylation levels of Akt and MAPK in tumours treated intraperitoneally with daily (left) or every 4 days (right) of stERAP-6 or unstapled original﻿ (ERAP). (**e**) Tumour growth by intravenous injection of stERAP-6 daily (left) or weekly (right) in KPL-3C orthotropic breast cancer xenograft mice. The tumour sizes represent the mean ± s.e.m. of each group (n = 5). *P < 0.05, **P < 0.01, and ***P < 0.001 via two-sided Student’s *t*-tests.

We next investigated the *in vivo* anti-tumour effect of stERAP-6 in KPL-3C orthotropic breast cancer xenografts in mice. Various concentrations of stERAP-6, unstapled original ERAP, or vehicle alone were intraperitoneally administered to mice at daily or every 4 days when tumours reached about 100 mm^3^ (Supplementary Fig. S3a). The percentage of tumour growth inhibition (TGI) was calculated as drug response (see Materials and Methods). Daily E2 treatment (6 μg per day) induced the time-dependent growth of KPL-3C tumours. Intraperitoneal administration of stERAP-6 at 1.4 and 14 mg kg^−1^ once a day for 28 days caused a significant inhibition of E2-induced tumour growth (TGI: 101.6% and 95.0%, respectively, on day 28) and unstapled original ERAP (TGI: 94.2% and 94.5%, respectively, on day 28; Fig. 3a). Treatment with stERAP-6 at 1.4 and 14 mg kg^−1^ every 4 days resulted in significant inhibition of E2-induced tumour growth (TGI: 103.7% and 103.3%, respectively, on day 28), whereas unstapled original ERAP treatment at 1.4 and 14 mg kg^−1^ every 4 days affected inhibition of E2-induced tumour growth less (TGI: 36.9% and 40.3%, respectively, on day 28; Fig. 3a). These results suggest that stERAP-6 possesses *in vivo* long-lasting suppressive activity. Importantly, no major morphological changes or body weight loss were observed in mice even though they received high doses of stERAP (14 mg kg^−1^) daily for 28 days (Supplementary Fig. S3b,c), although pulmonary inflammatory changes occurred severely in the mice of no treatment group, which was not ameliorated only by the E2 treatment alone. However, the pulmonary inflammatory changes were substantially suppressed in those of E2 plus stERAP treated-mice.

To clarify the mechanisms of the *in vivo* anti-tumour effect of stERAP-6 or unstapled original ERAP, we first examined their effects on the BIG3-PHB2 complex formation in tumours. Co-immunoprecipitation experiments in tumours indicated that treatment with stERAP-6 every 4 days, but not with unstapled original ERAP, led to effective inhibition of the endogenous BIG3-PHB2 complex formation (Fig. 3b). Treatment with stERAP-6 every 4 days also led to the E2-dependent nuclear translocation of cytoplasmic PHB2 in tumours (Supplementary Fig. S3d). Subsequently, treatment with stERAP-6 every 4 days also significantly suppressed E2-induced expression of *TFF1* and *CCND1* and phosphorylation levels of Akt and MAPK in tumours, but treatment with unstapled original ERAP did not (Fig. 3c,d). Our results demonstrated that stERAP-6 had potent, sustained *in vivo* anti-tumour activity via inhibition of E2-dependent genomic and non-genomic ERα activation.

Most importantly, to glean clinical insight, we investigated anti-tumour effects upon intravenous administration with stERAP-6. Treatment of 0.1, 1, and 10 mg kg^−1^ stERAP-6 weekly for 35 days resulted in significant inhibition of E2-induced tumour growth (TGI: 50.8%, 72.4%, and 96.1% on day 35, respectively) without any body weight loss (Fig. 3e and Supplementary Fig. S3e). Daily treatment with stERAP-6 also led to inhibited tumour growth (TGI: 68.4%, 86.1%, and 92.1% on day 35, respectively; Fig. 3e). However, alanine-mutant stERAP-6 had no *in vivo* anti-tumour effect despite daily treatment (Supplementary Fig. S3f). Co-immunoprecipitation experiments with tumours indicated that daily and weekly stERAP-6 treatment effectively inhibited the formation of the endogenous BIG3-PHB2 complex (Supplementary Fig. S3g). Taken together, these findings suggestd that stERAP-6 exhibited sustained inhibition of BIG3-PHB2 complex formation in tumours, thereby releasing PHB2 and allowing suppression of both genomic and non-genomic ERα activation along with E2-induced breast cancer growth *in vivo*.

### Inhibitory effect of combination of stERAP-6 with anti-cancer drugs in breast cancer cell growth

**Figure 4:**
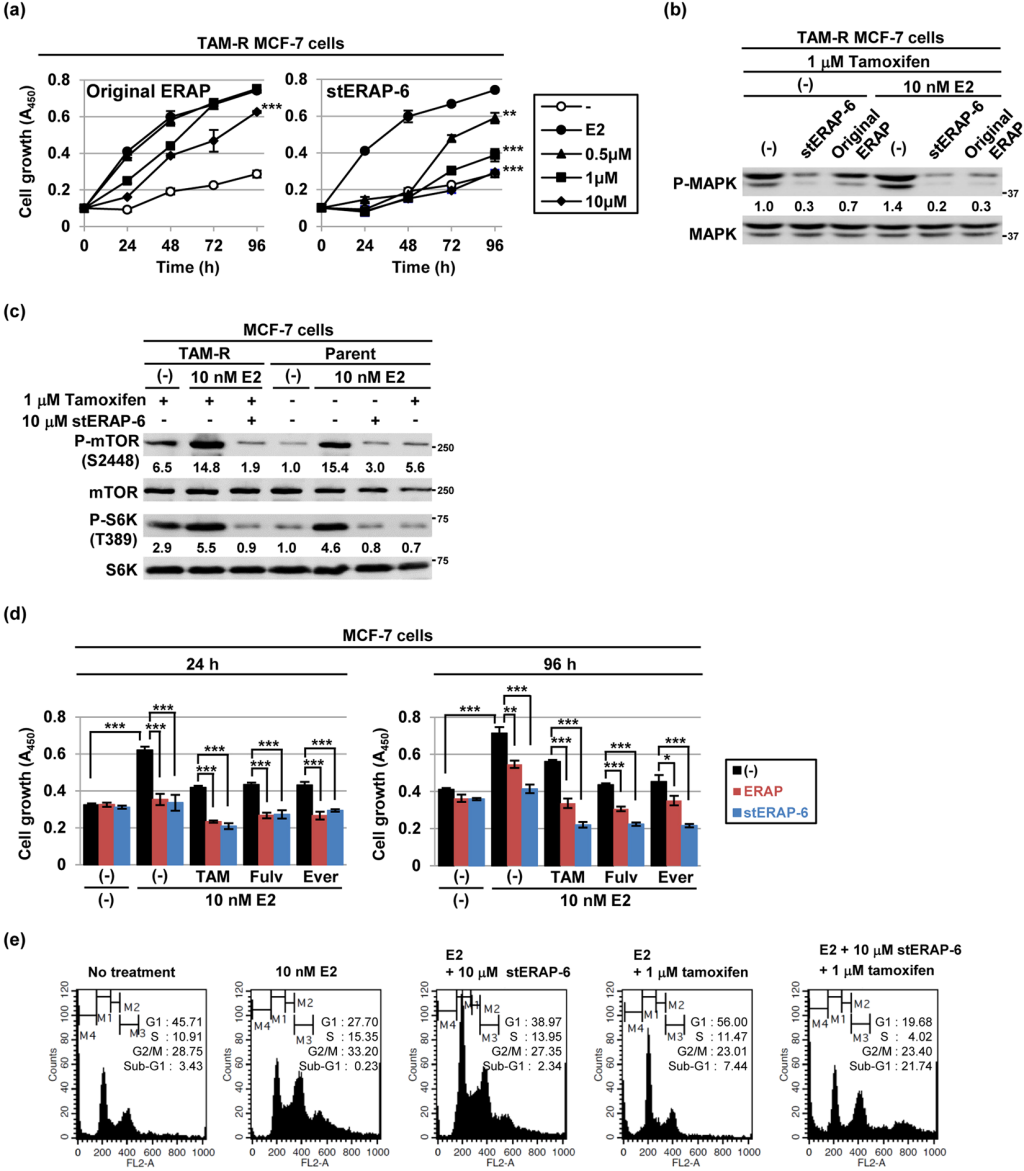
Stapled ERAP inhibits the E2-dependent growth of tamoxifen-resistant MCF-7 cells. (**a**) MTT assays evaluating the inhibitory effect of unstapled, original ERAP (left) and stERAP-6 (right) on the growth of tamoxifen-resistant (TAM-R) MCF-7 cells. TAM-R MCF-7 cells were treated with unstapled original ERAP or stERAP-6 in the presence of 10 nM E2 and 1 μM tamoxifen (TAM) at 0 h. The data represent the mean ± s.d. of three independent experiments. (**b**,**c**) Immunoblot analysis evaluating the phosphorylation levels of MAPK (**b**), mTOR, and S6K (**c**) in TAM-R MCF-7 cells. (**d**) The combined inhibitory actions of stERAP-6 and tamoxifen (TAM), fuluvestrant (Fulv) or everolimus (Ever) assessed via MTT assays. MCF-7 cells were treated for 24 h and 96 h with 10 nM E2 ± 10 μM stERAP-6, 10 μM unstapled original ERAP, 1 μM TAM, and a combination of stERAP-6 or unstapled original ERAP and 1 μM TAM, 2 μM Fulv or 0.5 μM Ever. The data represent the mean ± s.d. of three independent experiments. **P < 0.01 and ***P < 0.001 via two-sided Student’s *t*-tests. (**e**) Flow cytometric analyses evaluating the effect of stERAP-6 treatment on the cell cycle of MCF-7 cells.

Next, we examined the inhibitory effects of stERAP-6 in tamoxifen-resistant (TAM-R) MCF-7 cells. As shown in Fig. 4a, stERAP-6 treatment significantly reduced the growth of TAM-R MCF-7 cells in a dose-dependent manner in the presence of E2 and TAM for 96 h after treatment, whereas the inhibitory effect of unstapled original ERAP was reduced after 24 h treatment as previously described^12^. Subsequently, we examined the effects of stERAP-6 on the activation of the ERα-signalling pathway and found that stERAP-6 significantly inhibited the phosphorylation levels of p42/44 MAPK, which are factors responsible for TAM resistance (Fig. 4b), and of p-S6K (T389), a marker of downstream of PI3K and/or MAPK signalling pathway (Fig. 4c)^1, 13, 14^. Notably, stERAP-6 inhibited the phosphorylation level of p-S6K (T389) and p-mTOR (S2448) which is synergistically enhanced due to treatment of E2 and TAM in TAM-R cells (Fig. 4c).

Furthermore, we examined the effects of a combination of stERAP-6 (10 μM) and anti-breast cancer drugs on E2-induced cell proliferation. Treatment with a combination of stERAP-6 and TAM significantly suppressed E2-induced MCF-7 cell growth compared to stERAP-6 or TAM alone (Fig. 4d). Notably, a remarkable increase in the apoptotic (sub-G1) cell population of MCF-7 cells was observed after treatment with a combination of stERAP-6 and TAM (21.74%, treatment with stERAP-6 or TAM alone, 2.34% or 7.44%, respectively), although treatment with stERAP-6 or TAM alone suppressed cell growth by inducing a G1 arrest (Fig. 4d,e). More importantly, combination of stERAP-6 with fulvestrant, or everolimus caused synergistic inhibitory effects on the growth of breast cancer cells (Fig. 4d). Thus, remarkable synergistic reduction was observed after this combination treatment, suggesting that stERAP-6 enhanced the responsiveness of anti-breast cancer drugs to ERα-positive breast cancer cells.

## Discussion

PHB2 can not function as a potent co-repressor of ERα in breast cancer cells^15, 16^ despite its abundant expression. Our previous studies demonstrated that BIG3 preferentially interacts with PHB2 in the cytoplasm of cancer cells and sustains PHB2 in inactivate state even for E2 stimulation, resulting in E2-induced ERα constitutive activation of breast cancer cells^9, 10^. According to these findings, we established a therapeutic strategy for breast cancer utilizing the tumour-suppressive activity of PHB2 upon its release from BIG3 via the dominant negative peptide ERAP, which targets the BIG3-PHB2 interaction^10^. However, ERAP is difficult to use in clinical practice due to its limited stability. Therefore, in this study, we developed a chemically modified ERAP using stapling methods (stERAP) to improve the duration of its anti-tumour effects.

We synthesized a series of stapled α-helices of ERAP (Fig. 1a) and found that stERAP-2 and -6, which contained a hydrocarbon bridge between position 3 (167L) and position 7 (171T), showed the most marked enhancement of helical structure by CD analysis. We previously demonstrated the importance of 169D for BIG3 heterodimerization with PHB2^10^. This is probably why 169D is located centrally between 167L and 171T residues, which are incorporated by amino acid (X) for stapling to maintain α-helix formation. Notably, stERAP-4, which lacks two critical residues (169D and 173Q) for BIG3-PHB2 interaction, showed no dose-dependent inhibition in E2-dependent cell growth and exhibited an inhibitory effect in normal epithelial cell growth (Fig. 1b,c) due to the potential off-target effects. Accordingly, stERAP-2 and stERAP-6 possessed stabilized α-helices to specifically inhibit the interaction of BIG3-PHB2, thereby leading to E2-induced PHB2 suppressive activity, and karyopherin α-mediated nuclear-translocation of PHB2^17^, resulting in the complete suppression of E2 signalling pathways in breast cancer by binding to nuclear ERα.

Another important feature of stERAP-2 and stERAP-6 was its potential protease resistance, which is known to correlate with the degree of α-helical stabilization^18, 19^. *In silico* analysis predicted two chymotrypsin sites (L167 and L174) in the ERAP sequence (Supplementary Fig. S3h). Since stERAP-6 was constructed with the hydrocarbon staple between L167 and T171, this likely prevented local proteolytic degradation due to the disruption of the putative chymotrypsin site (L167), thereby exhibiting its long-lasting suppressive activity. However, it will be necessary to clarify the effect of these potential chymotrypsin sites in stERAP-2 and stERAP-6 via site-directed mutagenesis. Another critical feature of stERAP-2 and stERAP-6 was its enhanced cell permeability while lacking the membrane-permeable polyarginine sequence. We demonstrated that stERAP-6 bound to cytoplasmic PHB2 and thus translocated PHB2, along with stERAP-6, to the nucleus (Fig. 2f), although it still remains to be clarified how stapled ERAP entered cancer cells and the precise factors affect this property. Thus, hydrocarbon stapling ERAP may provide an effective strategy for therapeutic modulation of the BIG3-PHB2 interaction in E2-dependent breast cancer cells.

Furthermore, we here demonstrated that *in vivo* intraperitoneal treatment of stERAP-6 every 4 days (1.4 mg kg^−1^ and 14 mg kg^−1^, TGI: 103.7% and 103.3%, respectively) resulted in enhanced suppression of E2-induced tumour progression in mice bearing KPL-3C breast cancer cells compared with unstapled, original ERAP (1.4 mg kg^−1^ and 14 mg kg^−1^, TGI: 36.9% and 40.3%, respectively), further illustrating that stERAP enhances these suppression due to the prolonged inhibition of BIG3-PHB2 interaction via higher PHB2-binding affinity than unstapled original ERAP. Importantly, we demonstrated the anti-tumour effects of intravenous administration of stERAP-6 weekly for clinical application, which were comparable to our intraperitoneal administration data. Regarding the adverse effects of mice received high doses of stERAP-6 (14 mg kg^−1^) daily for 28 days, as expected, no morphological changes were observed in the heart, lung, liver, kidney, spleen, and pancreas of mice (Supplementary Fig. S3b) because BIG3 is hardly detectable in human organs, except in the brain^9^. Thus, to examine the possible adverse effects of stERAP-6 in the brain, we analyzed an *in vitro* blood-brain barrier (BBB) model using the BBB kit (see Supplementary Methods) and found that stERAP-6 barely penetrated into the central nervous system (Supplementary Fig. S3i), indicating that stERAP-6 has little ability to cross the BBB and induce brain damage. In addition, pulmonary inflammatory changes occurred severely in the mice of no treatment group, which was not ameliorated only by the E2 treatment alone. However, the pulmonary inflammatory changes were substantially suppressed in those of E2 plus stERAP treated-mice. These data suggest that pulmonary inflammatory lesion is an ancillary finding caused by tumour proliferation and invasion, although the precise mechanism governing pulmonary inflammation with tumour development remains unclear. The administration of tamoxifen or aromatase inhibitors frequently causes adverse events, including vasomotor and musculoskeletal symptoms, which correlate with the reduction of circulating E2 levels^20–23^. In comparison with these therapies, adverse effects caused by stERAP-6 are expected to be much lower because it has no effects on circulating E2 levels due to its mode of action.

Noteworthy, in combination with anti-breast cancer drugs such as tamoxifen, fulvestrant and everolimus, stERAP-6 more effectively suppressed cell growth compared with unstapled original ERAP (Fig. 4d). More importantly, we observed the synergistic inhibitory effect of stERAP with these anti-breast cancer drugs, suggesting that stapling leads to a sustained synergistic anti-tumour effect between these anti-breast cancer drugs, and stERAP-6 may be due to different mechanistic forces at play. In addition, stERAP-6 may provide long-lasting suppressive activity in ERα-negative breast cancer and endometrial cancer, because BIG3 and PHB2 are significantly overexpressed in some ERα-negative breast cancers^9^, and patients with endometrial cancer based on the RNAseq dataset from the Cancer Genome Atlas and Gene Expression Omnibus database (Supplementary Fig. S3j). In conclusion, our findings suggest that stERAP-6, either used solely or in combination with anti-breast cancer drugs such as tamoxifen, fulvestrant and everolimus may be a promising antitumour drug to suppress the growth of luminal-type, especially endocrine-resistant, breast cancers.

## Materials and Methods

### Ethical statement

All experiments were conducted according to protocols reviewed and approved by the Committee for Safe Handling of Living Modified Organisms (Permission number 28-5) and the Institutional Animal Care and Use Committee (Permission number 13135) in Tokushima University.

### Materials

A dominant-negative peptide (native ERAP; 11R-GGG-QMLSDLTLQLRQR), which was designed to specifically inhibit the BIG3-PHB2 interaction, was synthesized as described previously^10^. Tamoxifen, fulvestrant and everolimus were purchased from Sigma, LKT laboratories and Cell Signaling Technology, respectively. All other chemicals were of analytical grade.

### Stapled ERAP synthesis

Peptides were synthesized manually on Rink Amide AM resin (0.62 mmol amine g^−1^) using standard Fmoc solid-phase peptide synthesis. Briefly, Fmoc group cleavage was performed with 20% (v/v) piperidine in *N*,*N*-dimethylformamide (DMF) for 10 min at room temperature. The resin was washed with DMF, and Fmoc-protected amino acids (Fmoc-Xaa-OH) were coupled with *N*,*N*-diisopropylcarbodiimide (DIPCDI) and 1-hydroxybenzotriazole hydrate (HOBt·H_2_O) in DMF for 2 h at room temperature, followed by a wash with DMF.

Synthesis of stapled ERAP bearing the olefin was performed by ring-closing metathesis as shown in Supplementary Fig. S4a. After the construction of the protected peptides, the Fmoc group at the N-terminus was cleaved, and the obtained resin was treated with acetic anhydride (Ac_2_O) and pyridine in DMF for 30 min at room temperature for acetylation of the N-terminus amino group. N-Terminus acetylated peptide on solid support was treated with 40 mM solution of Hoveyda-Grubbs 2^nd^ catalyst in degassed *o*-dichlorobenzene for 10 min at 80 °C. The reaction was monitored by HPLC after cleavage of peptides from the resin. Deprotection of acid-labile protecting groups with concomitant release of peptides from resin was achieved using a cocktail of TFA/*m*-cresol/thioanisole/1,2-ethanedithiol/H_2_O (90:2.5:2.5:2.5:2.5 (v/v), 50 μL per 1 mg resin) at room temperature for 90 min. The resin-bound peptides were washed with dichloromethane and dried *in vacuo;* then the peptides were cleaved from the resin, purified by semi-preparative HPLC, and lyophilized.

Stapled ERAP without the olefin metathesis was synthesized via intramolecular amidation as shown in Supplementary Fig. S4b. N-Terminus capped peptide bound to resin was mixed with 20 mM solution of Pd(PPh_3_)_4_ in CHCl_3_/AcOH/*N*-methylmorpholine (92.5/5/2.5 (v/v)) and shaken at room temperature for 2 h. Obtained resin was treated with DIPCDI and HOBt·H_2_O in DMF at room temperature for 2 h. The resin was then washed with CH_2_Cl_2_ and dried. N-Terminus modification and cleavage was performed according to the standard Fmoc SPPS protocol described above.

### Cell proliferation assay

The MCF-7, KPL-3C, and MCF10A cell proliferation assays were performed using the Cell Counting Kit-8 (CCK-8, Dojindo) as described previously^10^. The data represent the mean ± SE of three independent experiments.

### Antibodies and immunoblot analyses

Immunoblot analyses were performed as described previously^10^. After SDS-PAGE, the membranes were blocked with 4% BlockAce solution (Dainippon Pharmaceutical) for 3 h and then incubated with antibodies against the following proteins: BIG3 (1:1,000)^10^; PHB2 (1:1,000, Abcam); Akt (1:1,000), phospho-Akt (S473) (587F11, 1:1,000); p44/42 MAPK (1:1,000), phospho-p44/42 MAPK (T202/Y204) (1:1,000); mTOR (1:1,000); phospho-mTOR (S2448) (1:1,000); S6K (1:1,000); phopho-S6K (T389) (1:1,000); α/β-tubulin (1:1,000) (Cell Signalling Technology); and LMNB1 (1:100, Sigma). After incubation with an HRP-conjugated secondary antibody (anti-mouse IgG-HRP, 1:5,000; anti-rat IgG-HRP; 1:5,000; or anti-rabbit IgG-HRP, 1:1,000; Santa Cruz Biotechnology) for 1 h, the blots were developed with an enhanced chemiluminescence (ECL) system (GE Healthcare) and scanned using an Image Reader LAS-3000 mini (Fujifilm). All the experiments were performed in triplicate at a minimum. Full-length images of immunoblots are shown in Supplementary Fig. S5.

### Immunoprecipitation

Immunoprecipitation analysis was performed as described previously^10^. The cell lysates were pre-cleared with normal IgG and rec-Protein G Sepharose 4B (Life Technologies) at 4 °C for 3 h. Then, the supernatants were incubated with 5 ng antibodies against BIG3 at 4 °C for 12 h. Next, the antigen-antibody complexes were precipitated with rec-Protein G Sepharose 4B at 4 °C for 1 h. The immunoprecipitated protein complexes were washed several times with the lysis buffer.

### Surface plasmon resonance interaction analysis

The Biacore 3000 (GE Healthcare) was used to analyze the interaction between stERAPs (stERAP-1 and -2) and PHB2. Recombinant PHB2 was bound to a CM5-sensor chip, and the indicated concentrations of stERAPs were used as analytes. Binding curves were displayed, and the dissociation (K_d_) rate constants were determined using BIAevaluation software (GE Healthcare).

### Circular dichroism (CD) spectra measurement

CD spectrum in the range of 185 to 265 nm was recorded at 25 °C in a 2 mm-path quartz cuvette. Protein concentration was 50 μg mL^−1^ in 10 mM sodium phosphate buffer (pH 7.0). Molar ellipticity [θ] was calculated^24^.

### Immunocytochemical staining measurement of PHB2 and HA-stERAP-6

MCF-7 cells were seeded at 5 × 10^4^ cells per well in 8-well chambers (Nunc Laboratory-Tek II Chamber Slide System, Thermo Fisher Scientific) for 48 h and then treated with E2 ± HA-stERAP for 24 h. The staining procedures were conducted as described previously^10^.

### Real-time PCR

The expression of the ERα target genes (*TFF1* and *CCND1*) was evaluated by real-time RT-PCR as described previously^10^. Each sample was normalized to the *β* *2-MG* mRNA content, and the results were expressed as the fold increase over untreated cells (set at 1.0). The data represent the mean ± SD of three independent experiments. The primers used were: *TFF1* 5′-GGCCTCCTTAGGCAAATGTT-3′ and 5′-CCTCCTCTCTGCTCCAAAGG-3′; *CCND1* 5′-CAGAAGTGCGAGGAGGAGGT-3′ and 5′-CGGATGGAGTTGTCGGTGT-3′; and *β* *2-MG* 5′-AACTTAGAGGTGGGGAGCAG-3′ and 5′-CACAACCATGCCTTACTTTATC-3′.

### Cell cycle assay

Cells were fixed in cold 70% ethanol, incubated with 20 µg mL^−1^ propidium iodide (Sigma) and 1 mg mL^−1^ ribonuclease A (Sigma), and analysed by flow cytometry using a FACSCalibur with CellQuest software (BD).

### *In vivo* tumour growth inhibition assays

KPL-3C cell suspensions (1 × 10^7^ cells per mouse) were mixed with an equal volume of Matrigel (Corning) and injected (200 μL total) into the mammary fat pads of 6-week-old female BALB/c nude mice (Charles River Laboratories). The mice were housed in a pathogen-free isolation facility with a 12 h light/dark cycle and were fed rodent chow and water *ad libitum*. The tumours developed over a period of a few days, reaching sizes of approximately 100 mm^3^ [calculated as 1/2 × (width × length^2^)], upon injection of 6 μg per day of E2 solution to the neck skin. For intraperitoneal administration, the mice were randomized into 10 treatment groups (five animals per group): (1) no treatment; (2) 6 μg per day E2; (3) E2 + 1.4 mg kg^−1^ day^−1^ ERAP daily; (4) E2 + 1.4 mg kg^−1^ day^−1^ ERAP every 4 days; (5) E2 + 14 mg kg^−1^ day^−1^ ERAP daily; (6) E2 + 14 mg kg^−1^ day^−1^ ERAP every 4 days; (7) E2 + 1.4 mg kg^−1^ day^−1^ stERAP-6 daily; (8) E2 + 1.4 mg kg^−1^ day^−1^ stERAP-6 every 4 days; (9) E2 + 14 mg kg^−1^ day^−1^ stERAP-6 daily; and 10() E2 + 14 mg kg^−1^ day^−1^ stERAP-6 every 4 days. For intravenous administration, the mice were randomized into 10 treatment groups (five animals per group): (1) no treatment; (2) 6 μg per day E2; (3) E2 + 0.1 mg kg^−1^ day^−1^ stERAP-6 daily; (4) E2 + 1 mg kg^−1^ day^−1^ stERAP-6 daily; (5) E2 + 10 mg kg^−1^ day^−1^ stERAP-6 daily; (6) E2 + 0.1 mg kg^−1^ day^−1^ stERAP-6 weekly; (7) E2 + 1 mg kg^−1^ day^−1^ stERAP-6 weekly; (8) E2 + 10 mg kg^−1^ day^−1^ stERAP-6 weekly; (9) E2 + 1 mg kg^−1^ day^−1^ alanine-mutant stERAP-6 daily; and (10) E2 + 10 mg kg^−1^ day^−1^ alanine-mutant stERAP-6 daily. E2 was delivered via the application of a solution to the neck skin as previously noted.

The tumour volume was measured with calipers, after which time the mice were sacrificed and tumours excised. TGI was calculated according to the formula: [1 − (T − T0)/(C − C0)] × 100, where T and T0 are the mean tumour volumes at day 28 or 35 and day 1, respectively, for the experimental group, and C and C0 are those for the E2-treated group. The organs (heart, lung, liver, kidney, pancreas, and brain) were also immediately removed. A portion of each tissue was fixed in 10% neutral formalin for histological examination, while the remaining tissue sample was frozen and preserved at −80 °C for subsequent western blot analysis. All the experiments were performed in accordance with the guidelines of the animal care and use facility at Tokushima University.

### Statistical analysis

Student’s *t*-tests were used to determine the significance of differences among the experimental groups. *P* < 0.05 was considered statistically significant.

### Data availability

The microarray data in Supplementary Fig. S1c and Table S1 were deposited in the Gene Expression Omnibus (GEO, GSE87378). For detection of functional gene annotation clusters, we used DAVID 6.7 (http://david-d.ncifcrf.gov) and geneMANIA (http://genemania.org/). The *BIG3* expression in patients with endometrial cancer referenced in Supplementary Fig. S1j were assessed from the GEO dataset (GSE17025) and TCGA dataset (http://cancergenome.nih.gov/). All the other data supporting the findings of this study are available within the article and its Supplementary Information Files or from the corresponding authors upon reasonable request.

## Electronic supplementary material

Supplementary Information
