# Supplementary material for: Stapled BIG3 helical peptide ERAP potentiates anti-tumour activity for breast cancer therapeutics
Source: Sci Rep. 2017 May 12;7:1821. doi: 10.1038/s41598-017-01951-6 (PMC5431889; doi:10.1038/s41598-017-01951-6)
Supplement: Supplementary file 1 — Supplementary Information [file 41598_2017_1951_MOESM1_ESM.pdf]

Supplementary Information

Stapled BIG3 helical peptide ERAP potentiates antitumor activity for breast cancer therapeutics

Tetsuro Yoshimaru[1,†,*], Keisuke Aihara[2,†], Masato Komatsu[1], Yosuke Matsushita[1], Yasumasa Okazaki[3], Shinya Toyokuni[3], Junko Honda[4], Mitsunori Sasa[5], Yasuo Miyoshi[6], Akira Otaka[2], and Toyomasa Katagiri[1,*]

[1]Division of Genome Medicine, Institute for Genome Research, Tokushima University, Tokushima 770-8503, Japan; [2]Institute of Health Biosciences and Graduate School of Pharmaceutical Sciences, Tokushima University, Tokushima 770-8505, Japan; [3]Department of Pathology and Biological Responses, Nagoya University Graduate School of Medicine, Aichi 466-8550, Japan; [4]Department of Surgery, National Hospital Organization Higashitokushima Medical Center, Tokushima 779-0193, Japan; [5]Department of Surgery, Tokushima Breast Care Clinic, Tokushima 770-0052, Japan; [6]Department of Surgery, Division of Breast and Endocrine Surgery, Hyogo College of Medicine, Hyogo 663-8501, Japan. [†]These authors contributed equally to this work.

Supplementary Figures

(a)

Peptide	Concentration (μM)	% inhibition of cell growth: MCF-7 cells 24	48	72	96	MCF-10A cells 24	48	72	96 h
ERAP: 11R-GGG-QMLSDLTLQLRQR	0.5	0	0	13	2	0	0	1	0
	1	41	6	0	0	0	0	0	1
	10	100	48	61	61	0	0	0	0
stERAP-1 : QXLSDXTLQLRQR	0.5	0	21	32	59	0	6	12	4
	1	100	53	34	70	0	5	10	5
	10	100	99	64	81	0	6	12	6
stERAP-2 : QMXSDLXLQLRQR	0.5	38	51	40	63	1	8	11	4
	1	100	99	59	68	0	5	12	9
	10	100	100	79	77	2	4	6	8
stERAP-3 : QMLXDLTXQLRQR	0.5	11	22	16	46	3	5	6	7
	1	100	75	37	66	7	43	71	55
	10	100	99	90	79	12	55	71	57
stERAP-4 : QMLSXLTLXLRQR	0.5	0	16	13	13	0	0	8	3
	1	0	0	3	11	1	1	73	53
	10	62	16	45	65	12	44	73	54
stERAP-5 : QMLSDXTLQXRQR	0.5	18	20	42	55	9	54	72	55
	1	100	46	82	81	10	58	73	58
	10	100	100	86	86	7	53	71	56

(b)

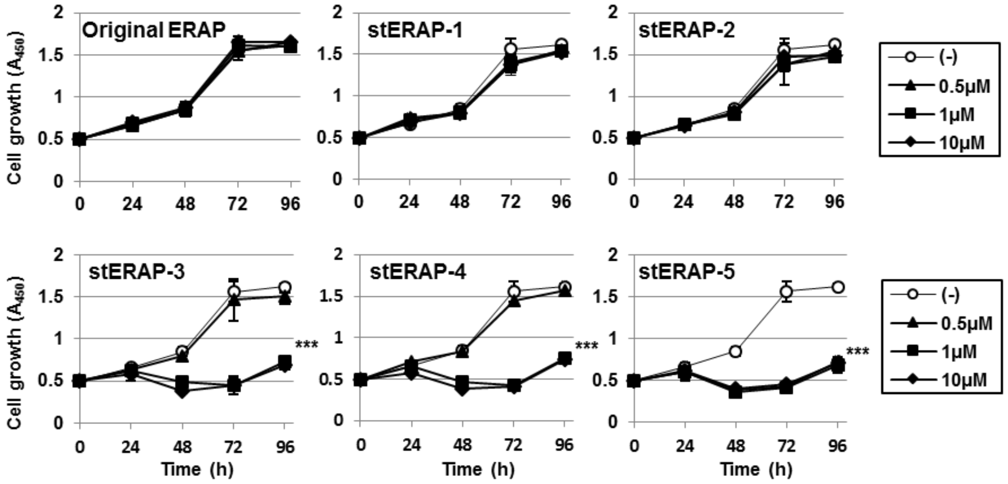

(c)

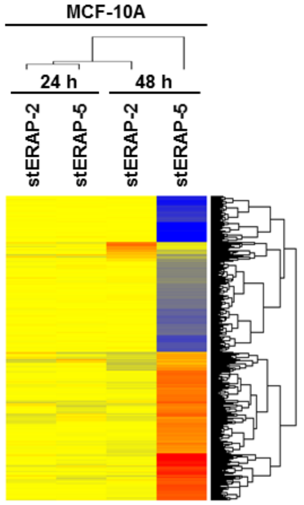

(d) **DAVID**

Term	Count	PValue	Bonferroni	Benjamini	FDR
cell adhesion	27	3.8E-10	1.3E-07	1.3E-07	5.2E-07
extracellular region part	42	8.3E-10	1.8E-07	1.8E-07	1.1E-06
signal	85	9.6E-09	3.3E-06	1.7E-06	1.3E-05
extracellular matrix	18	6.7E-08	2.3E-05	7.8E-06	9.2E-05
biological adhesion	34	1.7E-08	2.9E-05	9.8E-06	2.8E-05
signal peptide	85	1.3E-08	1.3E-05	1.3E-05	2.0E-05
cell motion	28	8.0E-09	1.4E-05	1.4E-05	1.4E-05
Secreted	52	1.9E-07	6.5E-05	1.6E-05	2.5E-04
calcium binding	12	2.4E-07	8.4E-05	1.7E-05	3.3E-04
inflammatory response	22	4.8E-08	8.4E-05	2.1E-05	8.1E-05
extracellular region	59	3.5E-07	7.7E-05	2.6E-05	4.5E-04
response to wounding	28	7.8E-08	1.4E-04	2.7E-05	1.3E-04
extracellular matrix	21	2.6E-07	5.8E-05	2.9E-05	3.4E-04
ectoderm development	17	1.1E-07	1.9E-04	3.1E-05	1.8E-04
epidermis development	16	2.3E-07	4.0E-04	5.7E-05	3.8E-04
proteinaceous extracellular matrix	19	1.7E-06	3.7E-04	7.4E-05	2.1E-03
extracellular space	29	1.4E-06	3.1E-04	7.7E-05	1.8E-03
epithelium development	16	3.2E-06	5.7E-03	7.1E-04	5.5E-03
glycoprotein	92	2.2E-05	7.6E-03	8.4E-04	3.0E-02
defense response	27	4.7E-06	8.2E-03	9.2E-04	7.9E-03
regulation of cell proliferation	31	6.6E-06	1.2E-02	1.2E-03	1.1E-02
cell migration	17	8.2E-06	1.4E-02	1.3E-03	1.4E-02
regulation of cell adhesion	12	1.1E-05	1.9E-02	1.6E-03	1.9E-02
epithelial cell differentiation	12	1.1E-05	1.94E-02	1.63E-03	1.88E-02

Gene MANIA

Feature	FDR	Coverage
extracellular matrix organization	1.1E−27	25/290
extracellular structure organization	1.1E−27	25/291
extracellular matrix	2.6E−22	20/208
extracellular matrix disassembly	9.6E−18	15/119
proteinaceous extracellular matrix	1.1E−14	13/110
hemidesmosome assembly	1.9E−12	7/12
cell junction assembly	1.9E−12	13/164
cell junction organization	5.3E−12	13/181
extracellular matrix part	5.7E−12	10/67
collagen catabolic process	2.8E−10	9/65
multicellular organismal catabolic proces	5.9E−10	9/71
cell−substrate junction assembly	1.4E−09	8/49
collagen	1.5E−09	7/28
extracellular matrix structural constitue	2.4E−09	8/53
collagen metabolic process	2.6E−09	9/86
multicellular organismal macromolecule	3.7E−09	9/90
multicellular organismal metabolic proce	6.3E−09	9/96
cell−substrate adhesion	1.3E−08	10/152
endoplasmic reticulum lumen	1.7E−07	9/140
cell−cell adhesion	1.9E−06	10/256
collagen fibril organization	5.3E−06	5/24
cell−matrix adhesion	1.3E−05	7/103
fibrillar collagen	1.5E−05	4/11
low−density lipoprotein particle binding	4.3E−05	1/14

Extracellular matrix organization pathway

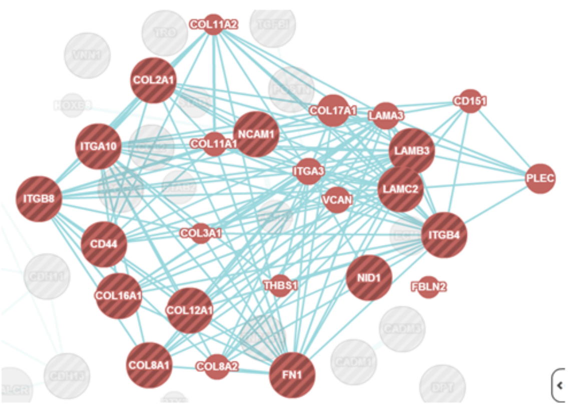

Collagen ; COL2A1, COL3A1, COL8A1, COL8A2, COL11A1, COL11A2, COL12A1, COL16A1, COL17A1
Integrin ; ITGA3, ITGA10, ITGB4, ITGB8
Laminin ; LAMA3, LAMB3, LAMC2
Fibronectin ; FN1

(e)

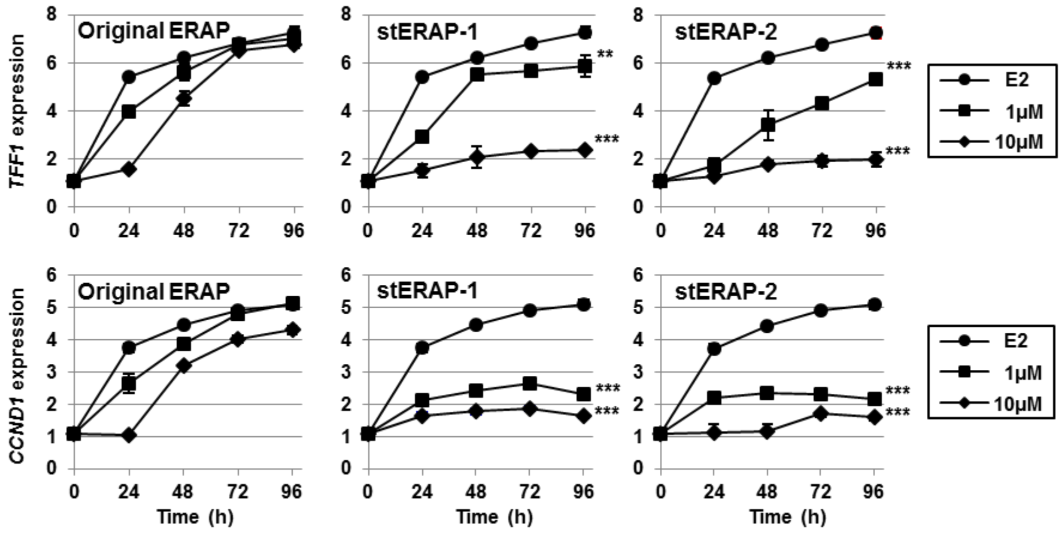

Supplementary Figure S1. Stapled ERAP suppresses the E2-dependent long-term responsiveness. (**a**) Percent growth inhibition of 10 nM E2-dependent MCF-7 cells and mammary epithelial MCF-10A cells by ERAP and its stapled analogs. (**b**) An MTT assay evaluating the inhibitory effects of stERAPs and unstapled, original ERAP on the growth of MCF-10A cells. Cells were given a single treatment at 0 h. These data represent the mean ± s.d. of three independent experiments. (**c**) Heat-map image representing 284 genes that were significantly up-regulated or down-regulated > 100-fold in stERAP-5 compared to stERAP-2 at 48 h post treatment. (**d**) Upper panel, gene annotation enrichment analysis based on DAVID. Lower panel, pathway analysis based on GeneMANIA software. (**e**) The duration of the inhibitory effects of stERAPs on ERα-target gene expression in MCF-7 cells. The results were expressed as the fold increase over untreated cells at 0 h (set at 1.0). The data represent the mean ± s.e.m. of three independent experiments. ***P* < 0.01 and ****P* < 0.001 via two-sided Student's *t*-tests.

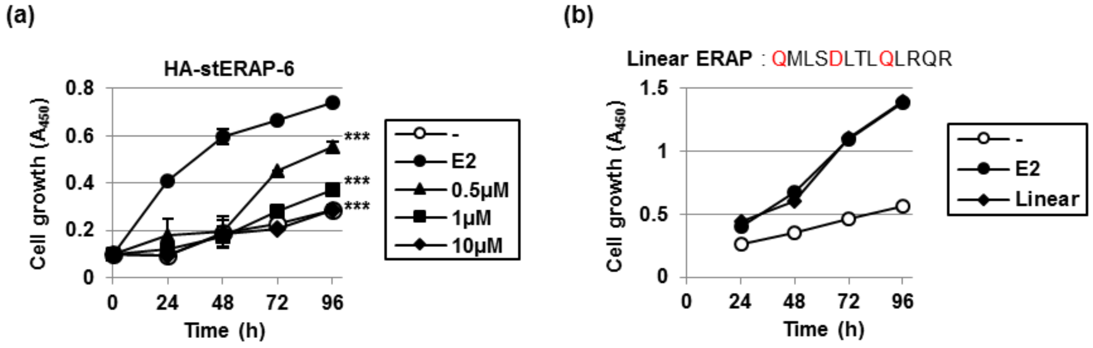

Supplementary Figure S2. HA-stERAP-6 suppresses the E2-dependent responsiveness. (**a**, **b**) MTT assays evaluating the inhibitory effects of HA-stERAP-6 (**a**) linear ERAP (**b**) on 10 nM E2-dependent growth of MCF-7 cells. Cells were given a single treatment at 0 h. These data represent the mean ± s.d. of three independent experiments. ****P* < 0.001 via a two-sided Student's *t*-test.

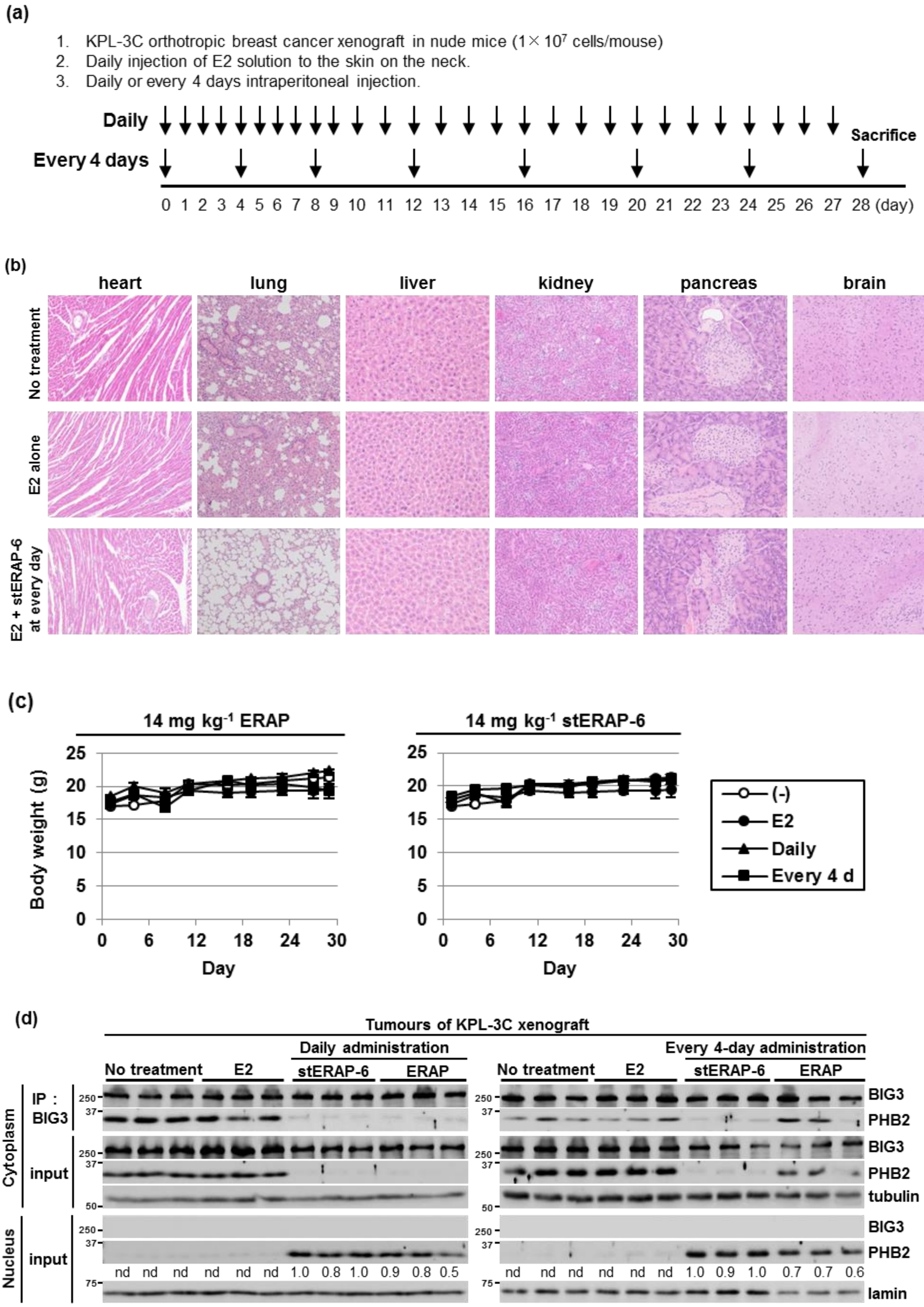
(a)
1. KPL-3C orthotropic breast cancer xenograft in nude mice (1× 10⁷ cells/mouse)
2. Daily injection of E2 solution to the skin on the neck.
3. Daily or every 4 days intraperitoneal injection.
Daily
Every 4 days
Sacrifice
0 1 2 3 4 5 6 7 8 9 10 11 12 13 14 15 16 17 18 19 20 21 22 23 24 25 26 27 28 (day)
(b)
heart
lung
liver
kidney
pancreas
brain
No treatment
E2 alone
E2 + stERAP-6 at every day
(c)
14 mg kg-1 ERAP
14 mg kg-1 stERAP-6
Body weight (g)
Day
(-)
E2
Daily
Every 4 d
(d)
Tumours of KPL-3C xenograft
Daily administration
Every 4-day administration
No treatment
E2
stERAP-6
ERAP
IP : BIG3
input
Cytoplasm
Nucleus
BIG3
PHB2
tubulin
lamin
nd nd nd nd nd nd 1.0 0.8 1.0 0.9 0.8 0.5
nd nd nd nd nd nd 1.0 0.9 1.0 0.7 0.7 0.6

(e)

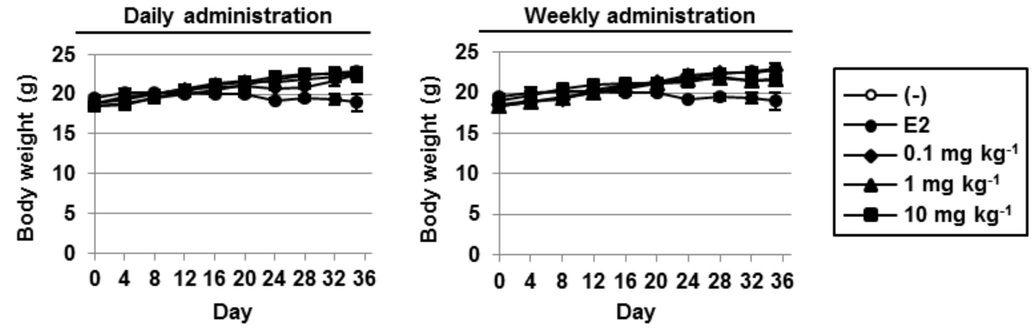

(f)

Alanine-mutant stERAP-6 : AMXSALXLALRQR

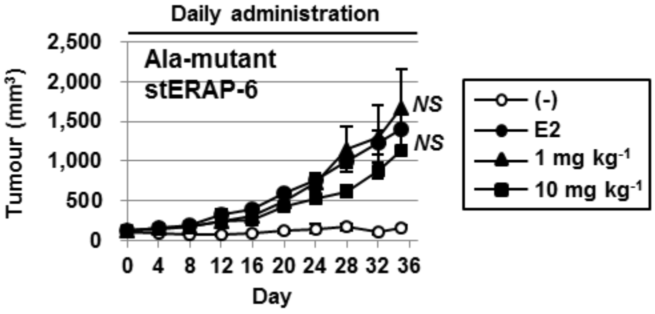

(g)

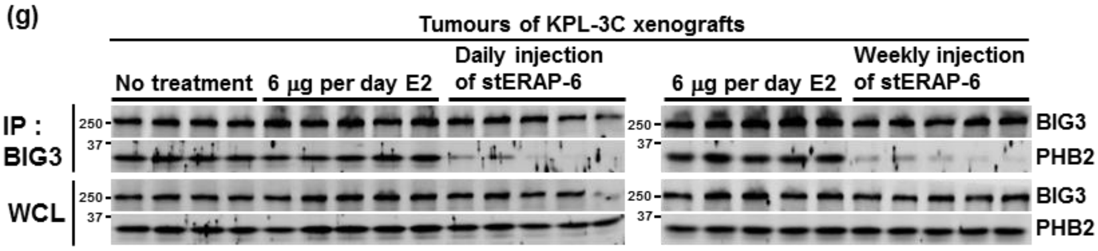

(h)

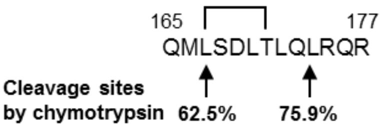

(i)

In vitro BBB permeability assay

		Papp (10^{-6} cm/sec)	Recovery Ratio (%)
stERAP-6	10 μM	0	0
	30 μM	0.177	0.01

(j)

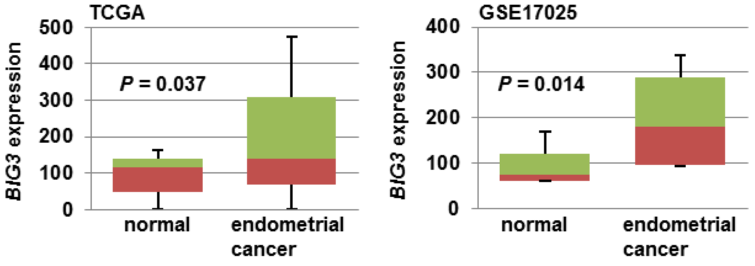

Supplementary Figure S3. StERAP-6 inhibits tumour growth in xenograft models of human ERα-positive breast cancer. (**a**) Schematic illustrations of *in vivo* experiments. (**b**) Representative HE staining of vital organs, including the heart, lung, liver, kidney, pancreas, and brain from mice treated daily with 14 mg kg^{-1} stERAP-6 for 28 days. (**c**) The body weights of the KPL-3C xenograft mice via intraperitoneally treatment daily or every 4 days with unstapled, original ERAP (left) and stERAP-6 (right) at 14 mg kg^{-1}. The body weights represent the mean ± s.d. of each group (n = 5). (**d**) Immunoblot analysis detecting the subcellular localization of BIG3 and PHB2 in tumours treated intraperitoneally daily (left) and every 4 days (right) with stERAP-6 or ERAP. α/β-tubulin (Tublin) and laminin B were used as loading controls for the cytoplasmic and nuclear fractions, respectively. (**e**) The body weights of the KPL-3C xenograft mice via intravenously treatment of stERAP-6 with daily (left) and weekly treatments (right). The body weights represent the mean ± s.d. of each group (n = 5). (**f**) Tumour growth by daily intravenous injection of alanine-mutant stERAP-6 in KPL-3C xenograft mice. The tumour sizes represent the mean ± s.e.m. of each group (n = 4). (**g**) Immunoblot analysis of the binding inhibition of BIG3-PHB2 in tumours treated intravenously daily (left) and weekly (right) with stERAP-6. (**h**) The potential chymotrypsin cleavage site of ERAP sequence using the Expasy PeptideCutter program. (**i**) Blood-brain barrier (BBB) permeability of stERAP-6. (**j**) mRNA expression of *BIG3* in patients with ERα-positive endometrial cancer based on the TCGA data set (left) and Gene Expression Omnibus database (GSE17025, right).

(a)

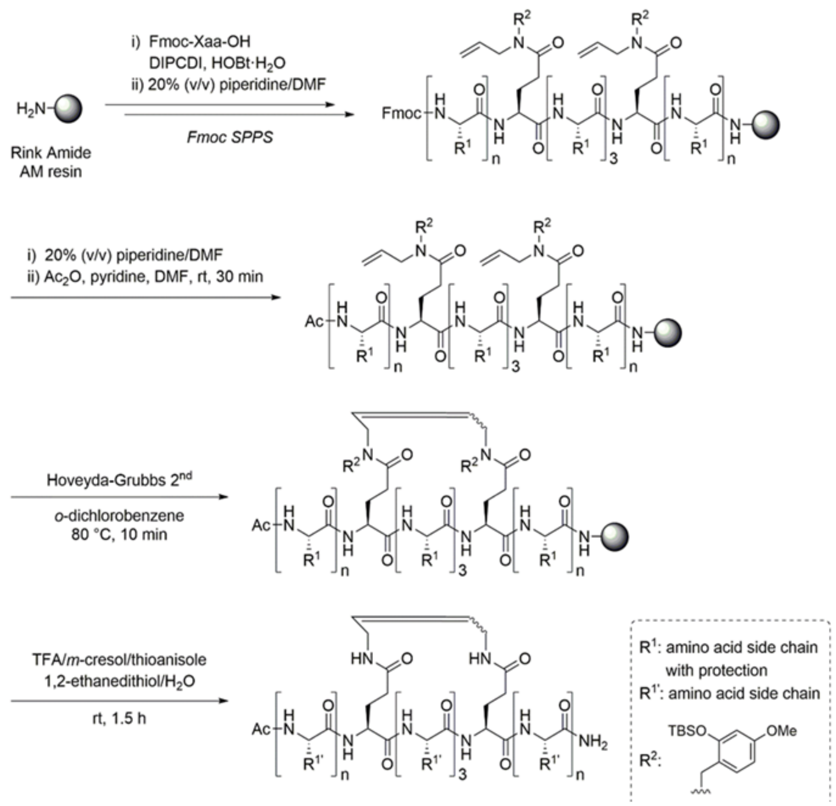

i) Fmoc-Xaa-OH
DIPCDI, HOBt·H2O
ii) 20% (v/v) piperidine/DMF
Fmoc SPPS
Rink Amide
AM resin
i) 20% (v/v) piperidine/DMF
ii) Ac2O, pyridine, DMF, rt, 30 min
Hoveyda-Grubbs 2nd
o-dichlorobenzene
80 °C, 10 min
TFA/m-cresol/thioanisole
1,2-ethanedithiol/H2O
rt, 1.5 h
R1: amino acid side chain
with protection
R1': amino acid side chain
R2:

(b)

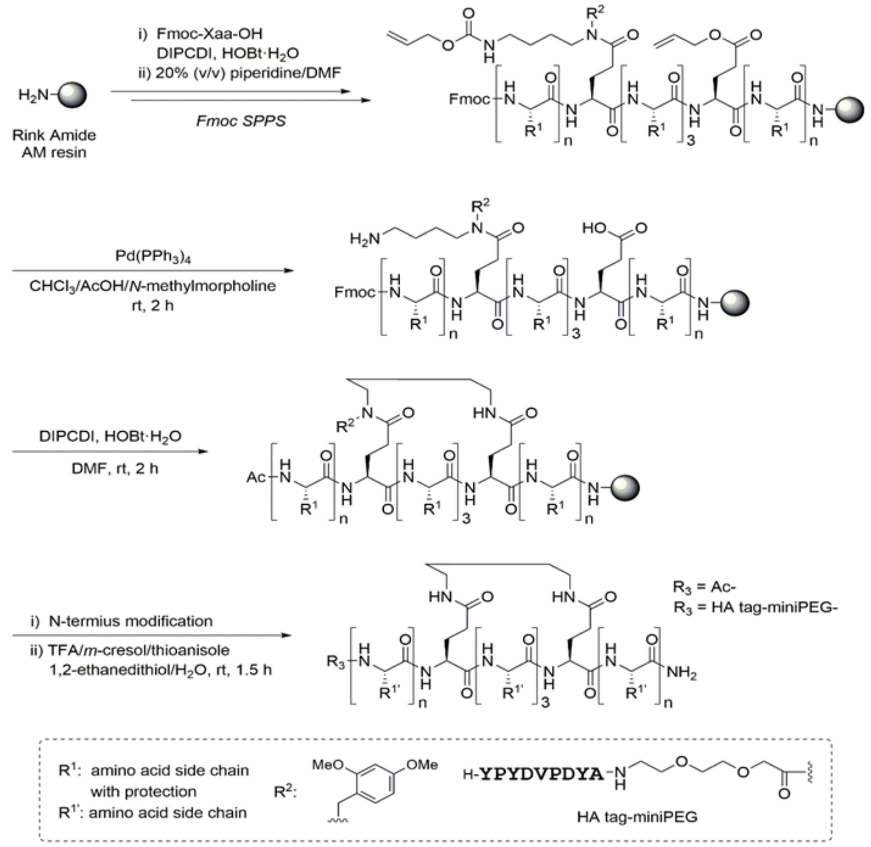

i) Fmoc-Xaa-OH
DIPCDI, HOBt·H2O
ii) 20% (v/v) piperidine/DMF
Fmoc SPPS
Rink Amide
AM resin
Pd(PPh3)4
CHCl3/AcOH/N-methylmorpholine
rt, 2 h
DIPCDI, HOBt·H2O
DMF, rt, 2 h
i) N-termius modification
ii) TFA/m-cresol/thioanisole
1,2-ethanedithiol/H2O, rt, 1.5 h
R3 = Ac-
R3 = HA tag-miniPEG-
R1: amino acid side chain
with protection
R1': amino acid side chain
R2:
H-YPYDVPDYA
HA tag-miniPEG

(c)

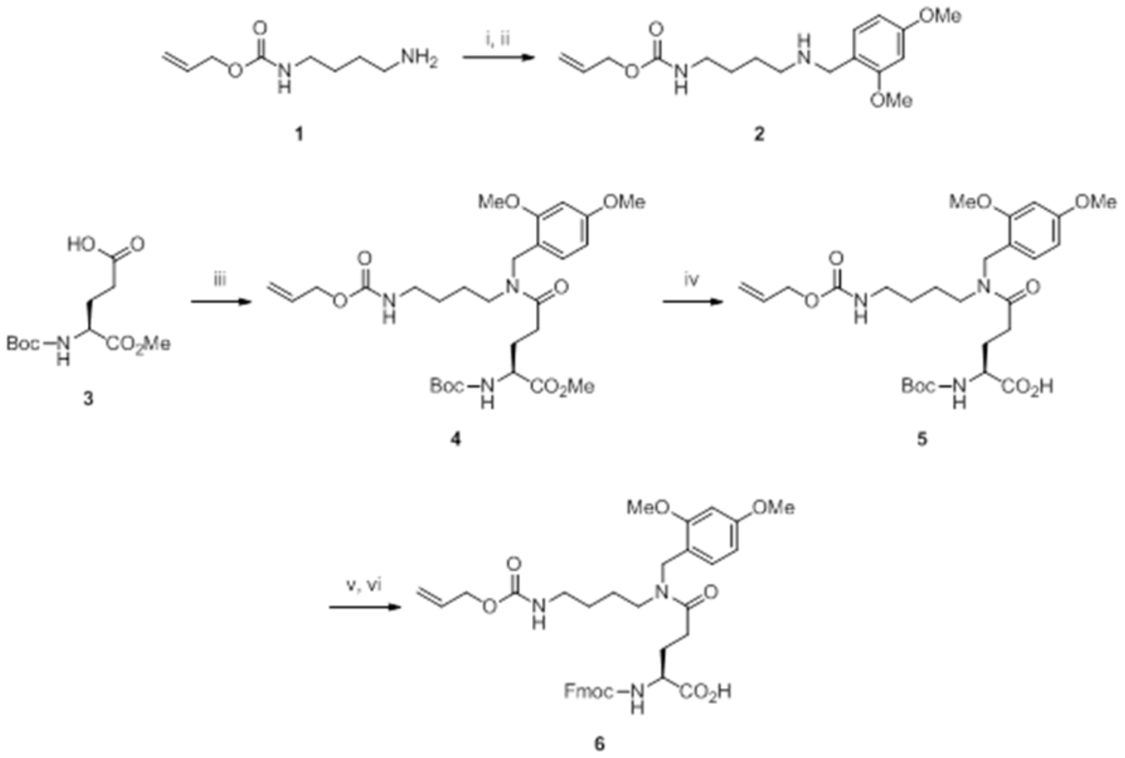

Supplementary Figure S4. Schematic illustrations of stapled ERAP synthesis. (**a**) Stapling synthesis of ERAP by ring-closing olefin metathesis. (**b**) Stapling synthesis of ERAP via intramolecular amidation. (**c**) Reagents and conditions of amino acid synthesis: (i) 2,4-dimethoxybenzaldehyde, AcOH, $MgSO_4$, and CH_2Cl_2; (ii) $NaBH_4$, MeOH, CH_2Cl_2; (iii) 2, EDC-HCl, DIPEA, CH_2Cl_2 (iv) $LiOH{\cdot}H_2O$, THF, MeOH, H_2O; (v) TBSOTf, 2,6-lutidine, CH_2Cl_2; and (vi) Fmoc-OSu, Na_2CO_3, THF, H_2O.

Figure. 1d

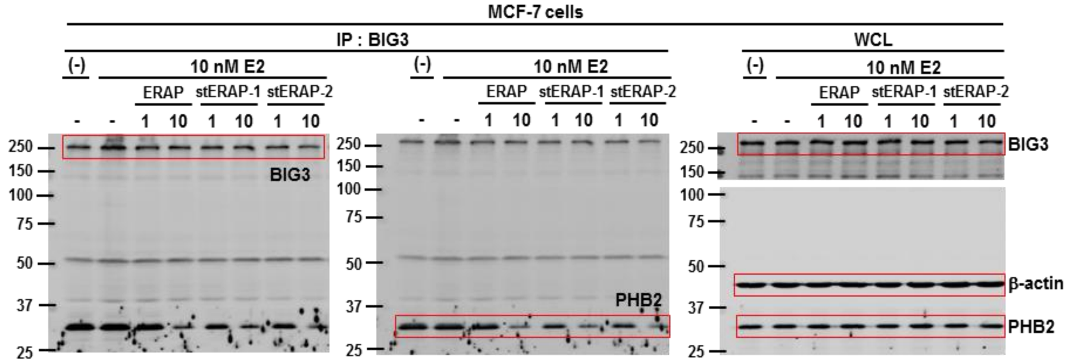

Figure. 2d

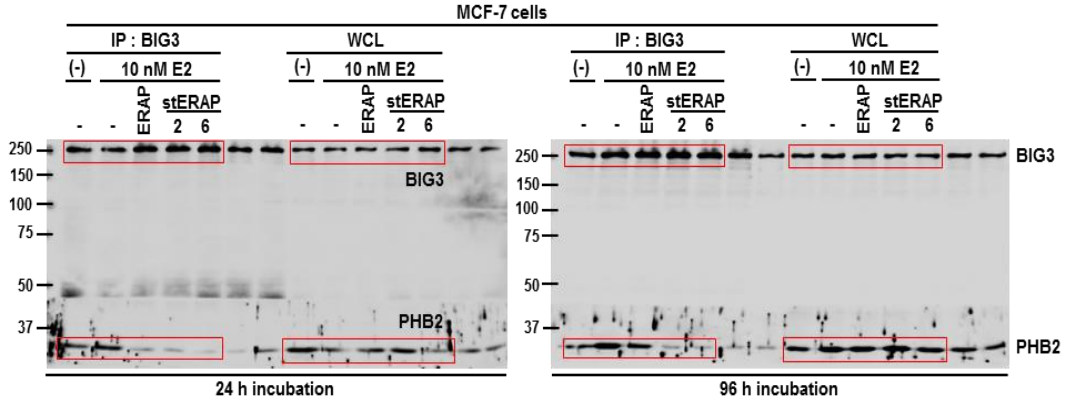

Figure. 3b

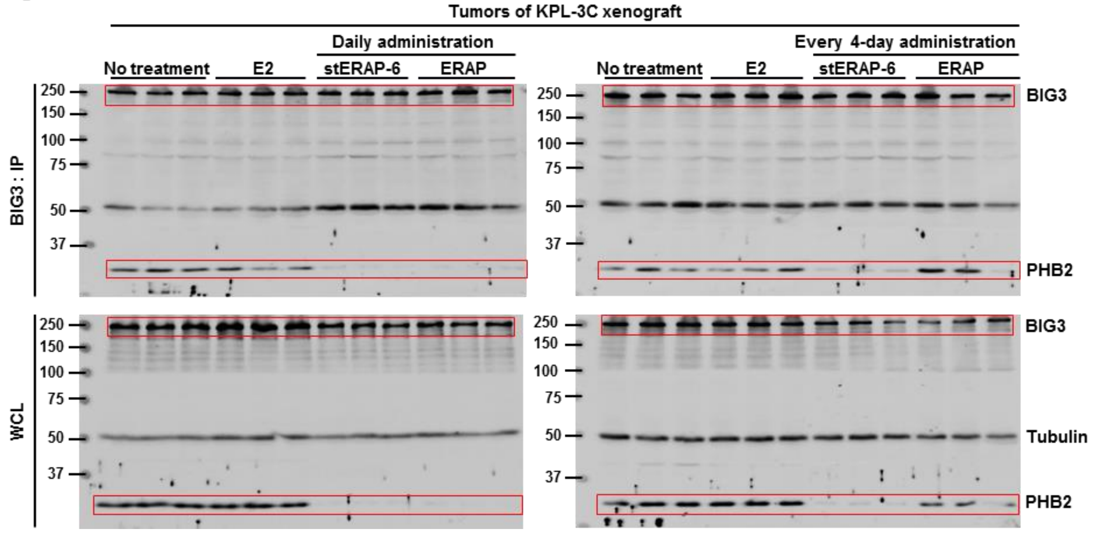

Figure. 3d

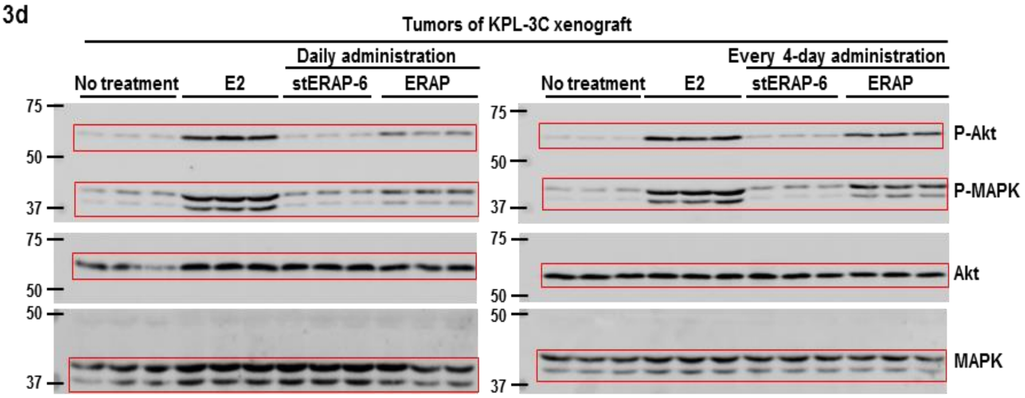

Figure. 4b

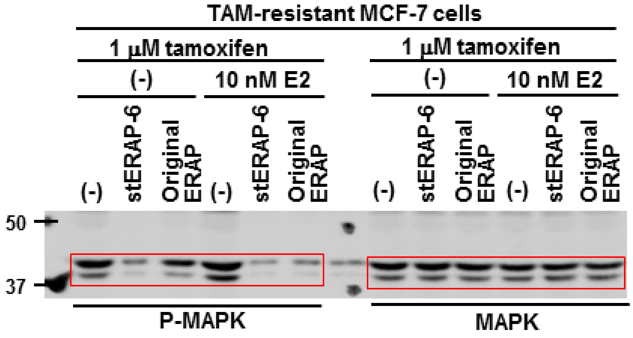

Figure. 4c

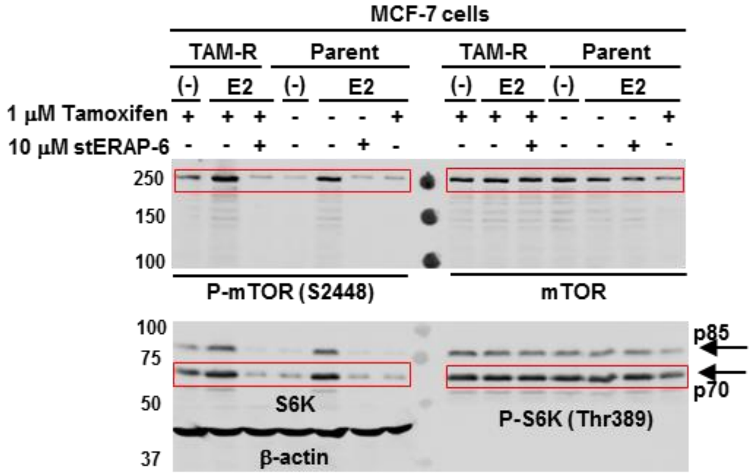

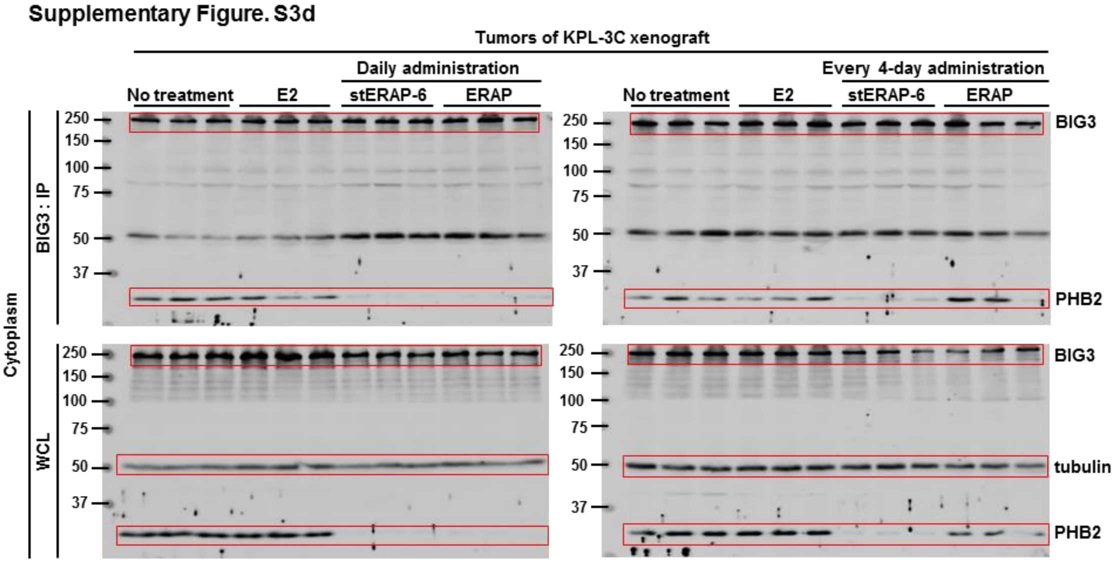

Supplementary Figure S5. Full-length images of immunoblots. Uncropped images of scanned immunoblots in Figures and Supplementary figures with size marker indications (kDa).

Supplementary Tables

Supplementary Table S1. Up- and down-regulated genes, more than 100-fold in stERAP-5 compared to stERAP-2 in 48 h treatments

Fold		Gene Symbol	Gene Name
3325	up	UCHL1	ubiquitin carboxyl-terminal esterase L1 (ubiquitin thiolesterase)
1710	up	KIF1A	kinesin family member 1A
1429	down	CSF3	colony stimulating factor 3 (granulocyte)
1366	down	TACSTD2	tumor-associated calcium signal transducer 2
1317	down	CADM3	cell adhesion molecule 3
1282	down	ARHGDIB	Rho GDP dissociation inhibitor (GDI) beta
1213	down	HSPA1A	heat shock 70kDa protein 1A
1197	down	AKR1C1	aldo-keto reductase family 1, member C1 (dihydrodiol dehydrogenase 1; 20-alpha (3-alpha)-hydroxysteroid dehydrogenase)
1184	down	KRT6A	keratin 6A
1161	up	BEX2	brain expressed X-linked 2
1117	down	OCIAD2	OCIA domain containing 2
1102	down	DCN	decorin
1085	up	GAL	galanin
1085	up	GSTM3	glutathione S-transferase mu 3
1030	down	MGP	matrix Gla protein
921	down	FN1	fibronectin 1
917	down	KRT5	keratin 5
880	down	MGMT	O-6-methylguanine-DNA methyltransferase
879	up	G0S2	G0/G1 switch 2
835	down	MT1M	metallothionein 1M
812	up	SNAR-D	steroidogenic acute regulatory protein
811	up	AMOT	fangiomotin
810	down	MLH1	mutL homolog 1, colon cancer, nonpolyposis type 2 (E. coli)
782	down	NALCN	sodium leak channel, non-selective
782	down	SLC16A3	solute carrier family 16, member 3 (monocarboxylic acid transporter 4)
748	down	SAA1	serum amyloid A1
717	down	S100A16	S100 calcium binding protein A16
716	down	C10orf116	chromosome 10 open reading frame 116
714	down	NNMT	nicotinamide N-methyltransferase
690	down	SAA2	serum amyloid A2
689	down	AOX1	aldehyde oxidase 1
686	down	GGT5	gamma-glutamyltransferase 5
686	up	BEX1	brain expressed gene 1
681	down	SPTLC3	serine palmitoyltransferase, long chain base subunit 3
679	down	CASP1	caspase 1, apoptosis-related cysteine peptidase (interleukin 1, beta, convertase)
674	up	SNAR-G2	small ILF3/NF90-associated RNA G2
668	up	SNAR-F	small ILF3/NF90-associated RNA E A
668	down	PTGFR	prostaglandin F receptor (FP)
666	down	CASP4	caspase 4, apoptosis-related cysteine peptidase
660	down	ANPEP	alanyl (membrane) aminopeptidase
621	down	CXCL1	chemokine (C-X-C motif) ligand 1 (melanoma growth stimulating activity, alpha)
609	up	ARMCX6	armadillo repeat containing, X-linked 6
600	down	C3	complement component 3
590	up	NEFM	neurofilament, medium polypeptide
586	up	DPYSL5	dihydropyrimidinase like 5
584	down	S100A6	S100 calcium binding protein A6
583	down	TM4SF1	transmembrane 4 L six family member 1
583	down	IRX1	iroquois homeobox 1
582	down	AKR1C3	aldo-keto reductase family 1, member C3 (3-alpha hydroxysteroid dehydrogenase, type II)
576	down	GPR110	G protein-coupled receptor 110
563	down	PPL	periplakin
559	down	MT1E	metallothionein 1E
543	up	IGF2BP1	insulin like growth factor 2 mRNA binding protein 1
543	down	CD44	CD44 molecule (Indian blood group)
541	down	IPW	imprinted in Prader-Willi syndrome (non-protein coding)
538	down	KRT14	keratin 14
535	up	SNAR-G1	small ILF3/NF90-associated RNA G1

Fold		Gene Symbol	Gene Name
535	up	LONRF2	LON peptidase N-terminal domain and ring finger 2
531	down	JPH2	junctophilin 2
526	down	SERPINB5	serpin peptidase inhibitor, clade B (ovalbumin), member 5
526	up	SNAR-H	small ILF3/NF90-associated RNA E
496	down	ZG16B	zymogen granule protein 16 homolog B (rat)
485	up	BCAT1	branched chain amino-acid transaminase 1, cytosolic
485	down	PSMB8	proteasome (prosome, macropain) subunit, beta type, 8 (large multifunctional peptidase 7)
484	down	SNRPN	small nuclear ribonucleoprotein polypeptide N
476	down	C19orf33	chromosome 19 open reading frame 33
471	down	ROS1	c-ros oncogene 1 , receptor tyrosine kinase
469	up	RADIL	Ras association and DIL domains
465	down	MAL2	mal, T-cell differentiation protein 2 (gene/pseudogene)
464	down	DARC	Duffy blood group, chemokine receptor
462	down	TGFBI	transforming growth factor, beta-induced, 68kDa
460	down	S100A3	S100 calcium binding protein A3
457	down	COL12A1	collagen, type XII, alpha 1
449	down	COL8A1	collagen, type VIII, alpha 1
445	down	AREG	amphiregulin
444	down	DUSP23	dual specificity phosphatase 23
443	down	ABCC3	ATP-binding cassette, sub-family C (CFTR/MRP), member 3
434	down	CDH13	cadherin 13, H-cadherin (heart)
434	up	HOXD13	homeobox D13
426	down	EMP1	epithelial membrane protein 1
418	up	CALCA	calcitonin-related polypeptide alpha
417	down	SPOCK1	sparc/osteonectin, cwcv and kazal-like domains proteoglycan (testican) 1
416	up	PCDH8	protocadherin 8
415	down	PRKCDBP	protein kinase C, delta binding protein
414	down	KCNK2	potassium channel, subfamily K, member 2
406	down	LAMB3	laminin, beta 3
398	down	PLEKHA6	pleckstrin homology domain containing, family A member 6
398	down	KCNN4	potassium intermediate/small conductance calcium-activated channel, subfamily N, member 4
395	down	SLC43A3	solute carrier family 43, member 3
392	down	TSTD1	thiosulfate sulfurtransferase (rhodanese)-like domain containing 1
389	up	C3orf14	chromosome 3 open reading frame 14
372	up	NEFL	neurofilament, light polypeptide
371	up	FAM101B	family with sequence similarity 101, member B
367	down	SLPI	secretory leukocyte peptidase inhibitor
360	up	NID1	nidogen 1
358	down	CCL2	chemokine (C-C motif) ligand 2
357	up	CADM1	cell adhesion molecule 1
348	up	ANKRD19P	ankyrin repeat domain 19, pseudogene
345	up	NPTX1	neuronal pentraxin I
343	down	FMO3	flavin containing monooxygenase 3
341	up	IGDCC3	immunoglobulin superfamily, DCC subclass, member 3
337	down	MLKL	mixed lineage kinase domain-like
326	down	MYOF	myoferlin
324	up	C11orf96	chromosome 11 open reading frame 96
315	up	SULT4A1	sulfotransferase family 4A member 1
314	down	FAP	fibroblast activation protein, alpha
314	down	TNS4	tensin 4
309	up	SNAR-A3	small ILF3/NF90-associated RNA A3
308	down	BIRC3	baculoviral IAP repeat containing 3
307	down	EMR2	egf-like module containing, mucin-like, hormone receptor-like 2
303	down	ADH1C	alcohol dehydrogenase 1C (class I), gamma polypeptide
300	down	S100A2	S100 calcium binding protein A2
299	up	PRDM13	PR domain containing 13

Continued on the next page

Fold		Gene Symbol	Gene Name
296	down	ITGB4	integrin, beta 4
290	down	PKP3	plakophilin 3
289	down	DKK1	dickkopf 1 homolog (Xenopus laevis)
275	down	ITGA10	integrin, alpha 10
271	up	DACH1	dachshund family transcription factor 1
271	up	FOXG1	forkhead box G1
270	down	LGALS1	lectin, galactoside-binding, soluble, 1
268	down	IFI44	interferon-induced protein 44
262	down	PLP2	proteolipid protein 2 (colonic epithelium-enriched)
254	down	GSTT2	glutathione S-transferase theta 2
250	down	CDCP1	CUB domain containing protein 1
250	down	CALHM2	calcium homeostasis modulator 2
249	down	DNAJC15	DnaJ (Hsp40) homolog, subfamily C, member 15
247	up	CA2	carbonic anhydrase II
247	down	HSD11B1	hydroxysteroid (11-beta) dehydrogenase 1
246	down	AKR1CL1	aldo-keto reductase family 1, member C-like 1
245	down	CSRP1	cysteine and glycine-rich protein 1
244	down	RAET1E	retinoic acid early transcript 1E
240	down	MIR100HG	mir-100-let-7a-2 cluster host gene (non-protein coding)
240	up	MTAP	methylthioadenosine phosphorylase
235	up	C7orf29	chromosome 7 open reading frame, humanC7orf29
230	down	SFN	stratifin
226	up	POU3F2	POU domain, class 3, transcription factor 2
225	up	TESC	tescalcin
224	down	ABCA12	ATP-binding cassette, sub-family A (ABC1), member 12
224	up	LRRC34	leucine rich repeat containing 34
223	up	HOXB5	homeobox B5
221	down	S100A8	S100 calcium binding protein A8
221	up	TRO	trophinin
219	up	FAM155B	family with sequence similarity 155 member B
216	down	CYP4B1	cytochrome P450, family 4, subfamily B, polypeptide 1
216	up	PRTFDC1	phosphoribosyl transferase domain containing 1
215	down	DNER	delta/notch-like EGF repeat containing
214	down	ANXA1	annexin A1
212	down	ABLIM3	actin binding LIM protein family, member 3
211	up	ZNF22	zinc finger protein 22
210	up	RPRML	reprimo-like
208	down	KDR	kinase insert domain receptor (a type III receptor tyrosine kinase)
208	down	KRT6C	keratin 6C
202	down	LAMC2	laminin, gamma 2
201	down	C2CD2	C2 calcium-dependent domain containing 2
201	down	COL16A1	collagen, type XVI, alpha 1
201	down	LTBR	lymphotoxin beta receptor (TNFR superfamily, member 3)
201	down	IL18	interleukin 18 (interferon-gamma-inducing factor)
198	down	C5orf38	chromosome 5 open reading frame 38
198	down	PID1	phosphotyrosine interaction domain containing 1
197	up	ALDH2	aldehyde dehydrogenase 2 family
195	down	TNFAIP6	tumor necrosis factor, alpha-induced protein 6
194	down	SHISA9	shisa homolog 9 (Xenopus laevis)
193	down	PLA2R1	phospholipase A2 receptor 1, 180kDa
193	down	IGFBP6	insulin-like growth factor binding protein 6
190	up	HOXB6	homeobox B6
189	down	IFI44L	interferon-induced protein 44-like
188	down	ITGB8	integrin, beta 8
187	down	OPLAH	5-oxoprolinase (ATP-hydrolysing)
187	up	RUNX3	runt-related transcription factor 3
185	down	CFH	complement factor H
185	down	MT1L	metallothionein 1L (gene/pseudogene)

Continued on the next page

Fold		Gene Symbol	Gene Name
185	down	PTGR1	prostaglandin reductase 1
183	up	HOXA11-AS1	HOXA11 antisense RNA
182	down	FAM198B	family with sequence similarity 198, member B
181	down	ARHGEF5	Rho guanine nucleotide exchange factor (GEF) 5
181	down	MLPH	melanophilin
181	up	POU4F1	POU domain, class 4, transcription factor 1
178	up	SERP2	stress-associated endoplasmic reticulum protein family member 2
177	up	BMP7	bone morphogenetic protein 7
176	down	LRRC3	leucine rich repeat containing 3
176	down	MT1B	metallothionein 1B
176	down	SCNN1A	sodium channel, nonvoltage-gated 1 alpha
174	up	COCH	cochlin
173	down	POSTN	periostin, osteoblast specific factor
172	down	PCDHB10	protocadherin beta 10
172	down	VSTM2L	V-set and transmembrane domain containing 2 like
169	down	FPR1	formyl peptide receptor 1
168	up	HOXD10	homeobox D10
168	down	IL20RB	interleukin 20 receptor beta
168	down	KRT17	keratin 17
166	down	RHOD	ras homolog gene family, member D
165	down	CFHR3	complement factor H-related 3
164	down	VNN1	vanin 1
163	up	ELOVL2	ELOVL fatty acid elongase 2
162	down	TNFSF14	tumor necrosis factor (ligand) superfamily, member 14
162	down	IRX2	iroquois homeobox 2
161	up	KIAA0408	KIAA0408
160	up	QPCT	glutaminyl-peptide cyclotransferase
159	down	ANXA8L2	annexin A8-like 2
158	up	CDKN2A	cyclin-dependent kinase inhibitor 2A
155	down	F2RL2	coagulation factor II (thrombin) receptor-like 2
155	up	CELF2	CUGBP, Elav-like family member 2
155	up	FBLL1	fibrillarin-like 1
153	up	KCNJ8	potassium channel, inwardly rectifying subfamily J, member 8
152	down	MIR205HG	MIR205 host gene (non-protein coding)
150	down	PEG10	paternally expressed 10
150	down	PLEK2	pleckstrin 2
149	down	MT1H	metallothionein 1H
149	up	C4orf49	chromosome 4 open reading frame, humanC4orf29
148	up	ONECUT2	one cut domain, family member 2
146	up	CNPY1	canopy FGF signaling regulator 1
146	down	ADH1A	alcohol dehydrogenase 1A (class I), alpha polypeptide
145	down	DSEL	dermatan sulfate epimerase-like
145	down	SAMD9L	sterile alpha motif domain containing 9-like
144	down	SNURF	SNRPN upstream reading frame
144	down	PCDHA11	protocadherin alpha 11
143	down	DPT	dermatopontin
142	up	ZIC3	zinc finger protein of the cerebellum 3
140	up	TSHZ3	teashirt zinc finger family member 3
139	down	IFIT2	interferon-induced protein with tetratricopeptide repeats 2
138	down	KRT16P2	keratin 16 pseudogene 2
137	down	VASN	vasorin
137	up	MPP2	membrane protein, palmitoylated 2
137	down	PARP12	poly (ADP-ribose) polymerase family, member 12
136	down	SQRDL	sulfide quinone reductase-like (yeast)
133	up	RBP1	retinol binding protein 1
131	down	EFEMP1	EGF containing fibulin-like extracellular matrix protein 1
130	down	PARP14	poly (ADP-ribose) polymerase family, member 14

Continued on the next page

Fold		Gene Symbol	Gene Name
130	down	CTGF	connective tissue growth factor
130	down	RIN1	Ras and Rab interactor 1
129	up	ZFHX4	zinc finger homeobox 4
128	down	PDZK1IP1	PDZK1 interacting protein 1
128	down	TRIM29	tripartite motif containing 29
128	down	AHNAK2	AHNAK nucleoprotein 2
128	up	EPDR1	ependymin related 1
127	up	NCAM1	neural cell adhesion molecule 1
126	down	H19	H19, imprinted maternally expressed transcript (non-protein coding)
126	down	IL8	interleukin 8
125	down	ANGPTL4	angiopoietin-like 4
125	up	FOXD3	forkhead box D3
125	down	TMEM173	transmembrane protein 173
123	down	MT1X	metallothionein 1X
123	up	ID4	inhibitor of DNA binding 4, dominant negative helix-loop-helix protein
122	down	C1S	complement component 1, s subcomponent
122	up	SLC35F1	solute carrier family 35 member F1
121	up	KRTAP19-1	keratin associated protein 19-1
121	up	PLAC1	placenta specific 1
120	down	DRAM1	DNA-damage regulated autophagy modulator 1
120	down	KLRC4	killer cell lectin-like receptor subfamily C, member 4
119	down	WWC3	WWC family member 3
118	up	COLEC11	rcollectin subfamily member 11
118	up	CYBA	cytochrome b-245, alpha polypeptide
117	up	TBX1	T-box 1
117	up	COL2A1	collagen, type II, alpha 1
116	down	NPR3	natriuretic peptide receptor C/guanylate cyclase C (atrionatriuretic peptide receptor C)
116	down	C1R	complement component 1, r subcomponent
115	down	IRX4	iroquois homeobox 4
113	down	ST8SIA1	ST8 alpha-N-acetyl-neuraminide alpha-2,8-sialyltransferase 1
113	down	TLR3	toll-like receptor 3
112	down	CFI	complement factor I
112	up	SMO	smoothened, frizzled class receptor
111	down	CASP5	caspase 5, apoptosis-related cysteine peptidase
111	up	ATF3	activating transcription factor 3
111	down	EBI3	Epstein-Barr virus induced 3
110	down	IFI16	interferon, gamma-inducible protein 16
110	up	SH2D3C	SH2 domain containing 3C
110	up	FOXN4	forkhead box N4
107	down	KRT83	keratin 83
107	down	PTRF	polymerase I and transcript release factor
107	up	ERC2	ELKS/RAB6-interacting/CAST family member 2
107	up	C15orf27	chromosome 10 open reading frame, human C15orf27
107	down	NT5E	5'-nucleotidase, ecto (CD73)
107	down	GSTT2B	glutathione S-transferase theta 2B (gene/pseudogene)
106	down	LSP1	lymphocyte-specific protein 1
106	down	TENC1	tensin like C1 domain containing phosphatase (tensin 2)
106	down	GNA15	guanine nucleotide binding protein (G protein), alpha 15 (Gq class)
105	down	CDH11	cadherin 11, type 2, OB-cadherin (osteoblast)
104	down	PYCARD	PYD and CARD domain containing
103	down	TGFBR2	transforming growth factor, beta receptor II (70/80kDa)
103	up	RRAGD	Ras-related GTP binding D
103	up	RNF182	ring finger protein 182
101	down	CLIC3	chloride intracellular channel 3
100	down	CAV1	caveolin 1, caveolae protein, 22kDa

Supplementary Table S2. Up- and down-regulated genes, more than 100-fold in stERAP-5 compared to stERAP-2 in 24 h treatments

Fold		Gene Symbol	Gene Name
81880	down	TDRD6	tudor domain containing 6
4199	up	psiTPTE22	TPTE pseudogene
409	down	ARL4A	ADP-ribosylation factor-like 4A
137	up	ZNF491	zinc finger protein 491

Supplementary Methods

Amino acids synthesis

For column chromatography, Silica Gel 60 N (spherical; neutral; particle size range: 63–210 nm; KANTO CHEMICAL, Tokyo, Japan) was employed. Mass spectra were recorded on a Waters MICROMASS® LCT PREMIER™ (ESI-TOF). NMR spectra were measured using a JEOL GSX300 spectrometer. For HPLC separation, a Cosmosil 5C_{18}-AR-II analytical column (4.6 × 250 mm, flow rate 1 mL/min, Nacalai Tesque, Kyoto, Japan) and a Cosmosil 5C_{18}-AR-II semi preparative column (10 × 250 mm, flow rate 3.0 mL/min, Nacalai Tesque) was employed, and eluting products were detected by UV at 220 nm. A solvent system consisting of 0.1% (v/v) TFA aqueous solution (solvent A) and 0.1% (v/v) TFA in MeCN (solvent B) was used for HPLC elution over 30 min. Optical rotation was determined on a JASCO P2200 polarimeter.

Amino acid synthesis was performed as shown in Supplementary Figure S4C.[1] Briefly, 2,4-dimethoxybenzaldehyde (781 mg, 4.70 mmol), $MgSO_4$ (2.26 g, 18.8 mmol), and AcOH (26.9 mL, 0.47 mmol) were added to a solution of allyl (4-aminobutyl) carbamate (**1**) (810 mg, 4.7 mmol) in MeOH (47 mL). The resulting mixture was stirred at room temperature for 3 h and filtered to remove

$MgSO_4$. The obtained reaction mixture was cooled to 0°C, and $NaBH_4$ (355 mg, 9.4 mmol) was added. The solution was warmed to room temperature and stirred for 1 h. The reaction mixture was quenched with 5% (w/v) aqueous solution of $KHSO_4$. The solution was basified with a saturated aqueous solution of $NaHCO_3$, and the mixture was extracted with EtOAc. The organic layer was washed with brine, dried over $MgSO_4$, and filtered. After concentration *in vacuo,* the crude product was purified by column chromatography ($CHCl_3$/MeOH = 30:1 (v/v)) and 1.32 g of amine (**2**) (allyl [4-{(2,4-dimethoxybenzyl)amino}butyl] carbamate, 4.09 mmol, 87%) was obtained as a pale yellow oil: ^{1}H NMR ($CDCl_2$, 300 MHz) δ = 1.42–1.58 (4H, m), 2.56 (2H, t, *J* = 6.7 Hz), 3.15 (2H, dt, *J* = 6.0 and 6.0 Hz), 3.67 (2H, s), 3.77 (3H, s), 3.78 (3H, s), 4.52 (2H, d, *J* = 5.5 Hz), 5.17 (1H, ddt *J* = 10.5, 1.5, and 1.5 Hz), 5.27 (1H, ddt *J* = 17.3, 1.5, and 1.5 Hz), 5.35 (1H, br s), 5.89 (1H, ddt, *J* = 17.3, 10.5, and 5.5 Hz), 6.40 (1H, dd, *J* = 8.1 and 2.4 Hz), and 6.43 (1H, d, *J* = 2.4 Hz), 7.09 (1H, d, *J* = 8.1 Hz); ^{13}C NMR ($CDCl_2$, 75 MHz) *5* = 27.4, 27.9, 41.0, 48.6, 48.9, 55.3, 55.4, 65.4, 98.6, 103.7, 117.4, 120.9, 130.5, 133.2, 156.4, 158.6, and 160.1; HRMS (ESI-TOF) *m/z calcd* for $C_{17}H_{27}N_2O_4$ ($[M + H]^+$): 323.1971, found: 323.1963.

The resultant amine (**2**) (1.22 g, 3.78 mmol), 1-(3-dimethylaminopropyl)-3-ethylcarbodiimide hydrochloride (EDC-HCl) (798 mg, 4.16 mmol), and diisopropylethylamine (DIPEA) were added to a solution of *N*-α-(*t*-butoxycarbonyl)-L-glutamic acid α-methyl ester (**3**) in 1,2-dichloroethane (18.9 mL) at 0°C. The mixture was stirred at room temperature for 5 h. After the addition of 5% (w/v) $KHSO_4$, the reaction mixture was extracted with EtOAc. The organic layer was washed with brine, dried over Na_2SO_4, filtered, and concentrated *in vacuo.* The crude product was purified by column chromatography (hexane/EtOAc = 1:2 (v/v)), and 1.62 g of amide (**4**) ((S)-Methyl-5-{(4-[{(allyloxy)carbonyl}amino]butyl)(2,4-dimethoxybenzyl)amino}-2-{(*tert*-butoxycarbonyl)amino}-5-oxopentanoate, 2.86 mmol, 76%) was obtained as a pale yellow oil: $[\alpha]^{19}_{D}$-5.33 (*c* 1.24, MeOH); ^{1}H NMR (DMSO-d_6, 300 MHz, 80°C) δ = 1.38 (9H, s), 1.27–1.53 (2H, m), 1.27–1.53 (2H, m), 1.46–1.92 (1H, m), 1.92–2.10 (1H, m), 2.42 (2H, dt, *J* = 4.5 and 6.6 Hz), 2.97 (2H, dt, *J* = 6.0 and 6.3 Hz), 3.19 (2H, br t, *J* = 7.0 Hz), 3.62 (3H, s), 3.76 (3H, s), 3.80 (3H, s), 3.94–4.14 (1H, m), 4.39 (2H, br s), 4.46 (2H, ddd, *J* = 5.5, 1.7, and 1.3 Hz), 5.16 (1H, ddt, *J* = 10.4, 1.8, and 1.3 Hz), 5.26 (1H, ddt, *J* = 17.2, 1.8, and 1.7 Hz), 5.90 (1H, ddt, *J* = 17.2, 10.4, and 5.5 Hz), 6.39–6.53 (1H, br m), 6.53–6.63 (1H,

br m), 6.70–6.92 (2H, br m), 6.96 (1H, br d, 7.9 Hz); ^{13}C NMR (DMSO-d_6, 75 MHz, 80°C) δ = 24.0, 25.2, 26.4, 26.5, 27.8, 28.3, 41.6, 44.4, 45.3, 46.1, 51.1, 53.1, 54.9, 55.1, 63.7, 77.9, 78.7, 98.4, 104.7, 116.3, 117.0, 128.0, 128.8, 133.5, 154.9, 155.5, 157.8, 159.5, 159.8, 171.0, 172.4; HRMS (ESI-TOF) *m/z calcd* for $C_{28}H_{43}N_3NaO_9$ ([M + Na]$^+$): 588.2897, found: 588.2902.

To a solution of amide 4 in THF (5 mL), LiOH·H_2O (91.8 mg, 2.18 mmol), MeOH (2.5 mL), and H_2O (2 mL) were added at 0°C, and the reaction mixture was stirred for 2 h. The reaction was quenched by the addition of 5% (w/v) $KHSO_4$, and the reaction mixture was extracted with EtOAc. The organic phase was washed with brine, dried over Na_2SO_4, filtered, and concentrated *in vacuo.* The residue was purified by column chromatography ($CHCl_3$/MeOH = 50:1–10:1 (v/v), containing 0.1% (v/v) AcOH) to yield 747 mg of carboxylic acid (**5**) ((S)-5-((4-(((Allyloxy)carbonyl)amino)butyl)(2,4-dimethoxybenzyl)amino)-2-((*tert*-butoxycarbonyl)amino)-5-oxopentanoic acid, 1.35 mmol, 92%) as a white powder: $[\alpha]^{18}_{D}$ -0.65 (*c* 0.950, MeOH); ^{1}H NMR (DMSO-d_6, 300 MHz, 80°C) δ = 1.39 (9H, s), 1.27–1.53 (2H, m), 1.27–1.53 (2H, m), 1.76–1.94 (1H, m), 1.94–2.13 (1H, m), 2.44 (2H, dt, J = 7.5 and 4.2 Hz), 2.99 (2H, dt, J = 6.3 and 6.1 Hz), 3.20 (2H, br t, J = 7.1 Hz), 3.76 (3H, s), 3.80 (3H, s), 3.89–4.08 (1H, m), 4.41(2H,

br s), 4.47 (2H, ddd, *J* = 5.4, 1.5, and 1.5 Hz), 5.16 (1H, ddt, *J* = 10.5, 1.7, and 1.5 Hz), 5.26 (1H, dt, *J* = 17.4, 1.7, and 1.5 Hz), 5.90 (1H, ddt, *J* = 17.4, 10.5, and 5.4 Hz), 6.38–6.53 (1H, br m), 6.56 (1H, br s), 6.66 (1H, br s), 6.78 (1H, br s), 6.98 (1H, br d, *J* = 8.1 Hz); ^{13}C NMR (DMSO-d_6, 75 MHz, 80°C) δ = 24.2, 25.3, 26.6, 26.7, 27.9, 28.6, 39.9, 41.8, 44.4, 45.4, 46.3, 53.1, 55.0, 55.2, 63.8, 77.8, 98.5, 104.8, 116.3, 117.1, 117.8, 128.1, 128.8, 133.5, 155.1, 155.6, 157.9, 159.6, 159.9, 171.3, 173.3; HRMS (ESI-TOF) *m/z* calcd for $C_{27}H_{41}N_3NaO_9$ ([M + Na]$^+$): 574.2741, found: 574.2740.

tert-Butyldimethylsilyl trifluoromethanesulfonate (TBSOTf) (1.04 mL, 4.5 mmol) and 2,6-lutidine (787 μL, 6.75 mmol) were added to a solution of Boc derivative 5 (621 mg, 1.13 mmol) in CH_2Cl_2 (11.3 mL) at 0°C. The reaction mixture was warmed to room temperature slowly and stirred for 4 h. The solution was concentrated *in vacuo* and then diluted with THF (8 mL). After the solution was neutralized with 2 M aqueous solution of NaOH (2 mL) at 0°C, 10% (w/v) Na_2CO_3 (8 mL) and Fmoc-OSu (572 mg, 1.7 mmol) were added to the solution. After 12 h stirring at room temperature, the mixture was acidified with 1 M aqueous solution of HCl and extracted with EtOAc. The organic layer was washed with brine, dried over Na_2SO_4, filtered, and concentrated. The residue

was purified by column chromatography ($CHCl_3$/MeOH = 50:1–10:1 (v/v), containing 0.1% (v/v) AcOH) to yield (**6**) ((S)-2-((((9H-fluoren-9-yl)methoxy)carbonyl)amino)-5-((4-(((allyloxy)carbonyl)amino)butyl)(2,4-dimethoxybenzyl)amino)-5-oxopentanoic acid, 680 mg, 1.01 mmol, 90%) as a white powder: $[\alpha]^{19}_{D}$ +8.38 (*c* 1.13, MeOH); ^{1}H NMR (DMSO-d_6, 300 MHz, 80°C) δ = 1.29–1.61 (4H, m), 1.87–2.02 (1H, m), 2.03–2.21 (1H, m), 2.45-2.56 (2H, m), 3.00 (2H, dt, *J* = 6.0 and 6.4 Hz), 3.23 (2H, br t, *J* = 6.6 Hz), 3.74 (3H, s), 3.79 (3H, s), 4.05–4.18 (1H, m), 4.22 (1H, t, *J* = 6.6 Hz), 4.31 (2H, d, *J* = 6.6 Hz), 4.43 (1H, br s), 4.48 (2H, ddd, *J* = 5.7, 1.7, and 1.5 Hz), 5.12 (1H, ddt, *J* = 10.2, 1.7, and 1.5 Hz), 5.27 (1H, ddt, *J* = 17.1, 1.7, and 1.7 Hz), 5.91 (1H, ddt, *J* = 17.1, 10.2, and 5.7 Hz), 6.42–6.52 (1H, br m), 6.56 (1H, d, *J* = 2.1 Hz), 6.80 (1H, br s), 6.91–7.10 (1H, br m), 7.31 (2H, t, *J* = 7.5 Hz), 7.40 (2H, t, *J* = 7.2 Hz), 7.70 (2H, br d, *J* = 7.2 Hz), 7.85 (2H, d, *J* = 7.5 Hz); ^{13}C NMR (DMSO-d_6, 75 MHz, 80°C) δ = 24.2, 25.3, 26.6, 28.6, 39.9, 41.8. 44.3, 45.3, 46.6, 53.4, 54.9, 55.1, 63.8, 65.6, 98.4, 104.7, 108.6, 116.3, 117.0, 117.7, 119.6, 120.9, 124.8, 126.6, 126.6, 126.8, 127.2, 128.2, 128.5, 128.9, 133.5, 139.2, 140.4, 140.4, 143.6, 143.6, 155.6, 155.6, 157.8, 159.6, 159.9, 171.2, 173.1; HRMS (ESI-TOF) *m/z* calcd for $C_{37}H_{43}N_3NaO_9$ ($[M + Na]^+$): 696.2897, found: 696.2928.

Cell lines and culture conditions. A human breast cancer cell line, MCF-7, and a mammary epithelial cell line, MCF-10A, were purchased from American Type Culture Collection (ATCC, Manassas, VA, USA). The KPL-3C cells were established, characterized, and kindly provided by Dr. Jun-ichi Kurebayashi (Kawasaki Medical School).[2] All cell lines were cultured as monolayers in an appropriate medium supplemented with 10% FBS. Cells were maintained at 37°C in an atmosphere of humidified air with 5% CO_2. The MCF-7 cells were seeded in 48-well plates (2×10^4 cells/mL), 6-well plates (3×10^5 cells/mL), or 10-cm dishes (2×10^6 cells/mL) in MEM (Life Technologies, Rockville, MD, USA) supplemented with 10% FBS (Nichirei Biosciences, Tokyo, Japan), 1% antibiotic/antimycotic solution (Life Technologies), 0.1 mM NEAA (Life Technologies), 1 mM sodium pyruvate (Life Technologies), and 10 ng/mL insulin (Sigma). KPL-3C cells were seeded in RPMI (Life Technologies) supplemented with 10% FBS and 1% antibiotic/antimycotic solution. MCF-10A cells were seeded in MEBM (Lonza, Walkersville, MD, USA) supplemented with SingleQuots Kit (BPE, hydrocortisone, hEGF, insulin, and gentamicin/amphotericin-B) and 100 ng/mL cholera toxin. For 17-estradiol (E2,

Sigma) stimulation in MCF-7 and KPL-3C cells, the medium was changed the next day to phenol red-free DMEM/F12 (Life Technologies) supplemented with FBS, antibiotic/antimycotic solution, NEAA, sodium pyruvate, and insulin. After 24 h, the cells were treated with 10 nM E2 ± peptides (e.g., ERAP or stapled ERAP).

Microarray analysis. Total RNA was purified with the NucleoSpin RNA II system (Takara-Clontech) according to the manufacturer's instructions. For RNA amplification and labeling, we used an Agilent Low-Input QuickAmp labeling kit (Agilent Technologies). Briefly, 100 ng of total RNA from each sample was amplified using T7 RNA polymerase with simultaneous Cy3-labeled CTP incorporation. Then, 600 ng of Cy3-labeled cRNA was fragmented, hybridized onto the Agilent Whole Human Genome Microarray 8 × 60K slide (Agilent Technologies), and incubated with rotation at 65°C for 18 h. The slides were washed and scanned using an Agilent Microarray scanner system in an ozone protection fume hood. The scanned image files containing the Cy3-fluorescence signals were extracted using the Agilent Feature Extraction (version 9.5, Agilent Technologies). The data were analyzed using GeneSpring (version 13.0). We

normalized the microarray data across all chips and genes by quantile normalization, and transformed the signal values to the median in all samples. To identify genes that were significantly altered, the mean signal intensity values in each analysis were compared.

Functional gene annotation clustering. DAVID 6.7 (refs. 3,4) and geneMANIA database, which is based on the Cytoscape plugin,[5] were approved to detect functional gene annotation clusters based on expression profiling by gene annotation enrichment analysis.

The morphological evaluation of xenograft model. To examine the morphological changes in each organ (heart, lung, liver, kidney, pancreas, and brain) of xenograft mice, the specimens were fixed in 10% phosphate-buffered formalin, embedded in paraffin, cut at 4 μm thickness and stained with hematoxylin and eosin. Registered pathologists (YO and ST) evaluated the morphological changes in vital organs of mice.

Prediction of chymotrypsin cleavage site of stERAP-6. Potential chymotrypsin (EC 3.4.21.1) cleavage site of stERAP-6 was predicted using the Expasy PeptideCutter program (http://ca.expasy.org/cgi-bin/peptidecutter/peptidecutter.pl).

***In vitro* blood-brain barrier (BBB) model.** To investigate the ability of ERAP and stERAP to penetrate into the central nervous system, the BBB kit (RBT24-H) was used as an *in vitro* BBB model (PharmaCo-Cell, Nagasaki, Japan) as previously described.[6,7] This BBB model was reconstructed using cultured primary rat brain microvascular endothelial cells and rat brain pericytes separated by a macroporous Millicel membrane (24 wells, pore size: 3.0 μm, Millipore, Bedford, MA, USA). Briefly, FITC-tagged stERAP-6, suspended in 0.2 mL assay medium (10 mM HEPES and 25 mM D-glucose in PBS), was added into the luminal side and incubated for 30 min. We collected the medium at the abluminal sides. The fluorescence intensity of FITC-stERAP-6 that permeated the abluminal sides was measured with a Micro-plate Reader Infinite 200 (Tecan, Mannedorf, Switzerland). The permeability coefficient (P*app*) was calculated using a formula provided by Pharmaco-Cell Company Ltd.

BIG3 expression in endometrial cancer. BIG3 expression in ERα-positive endometrial cancer were evaluated from publicly available The Cancer Genome Atlas (TCGA) RNA-seq V2 dataset (normal, 22 samples and endometrial tissue, 177 samples) and Gene Expression Omnibus database (GSE17025; normal, 12 samples and endometrial tissue, 80 samples).

Supplementary References

1. Hurevich, M. et al. Novel method for the synthesis of urea backbone cyclic peptides using new Alloc-protected glycine building units. *J. Pept. Sci.* **162**, 178–185 (2010).
2. Kurebayashi, J., Kurosumi, M. & Sonoo, H. A new human breast cancer cell line, KPL-3C, secretes parathyroid hormone-related protein and produces tumours associated with microcalcifications in nude mice. *Br. J. Cancer* **74**, 200–207 (1996).

3. Huang, D. W., Sherman, B. T. & Lempicki, R. A. Systematic and integrative analysis of large gene lists using DAVID Bioinformatics Resources. *Nat. Protoc.* **4**, 44–57 (2009).

4. Huang, D. W., Sherman, B. T. & Lempicki, R. A. Bioinformatics enrichment tools: paths toward the comprehensive functional analysis of large gene lists. *Nucleic Acids Res.* **37**, 1–13 (2009).

5. Montojo, J. et al. GeneMANIA: fast gene network construction and function prediction for cytoscape. *F1000Res.* **6**, 153; 10.12688/f1000research.4572.1 (2013).

6. Nakagawa, S. *et al.* A new blood-brain barrier model using primary rat brain endothelial cells, pericytes and astrocytes. *Neurochem. Int.* **54,** 253–263 (2009).

7. Pelisch, N. *et al.* Plasminogen Activator Inhibitor-1 Antagonist TM5484 Attenuates Demyelination and Axonal Degeneration in a Mice Model of Multiple Sclerosis. *PLoS One* **10,** e0124510; 10.1371/journal.pone.0124510 (2015).
